# Transcriptome analyses of liver in newly-hatched chicks during the metabolic perturbation of fasting and re-feeding reveals THRSPA as the key lipogenic transcription factor

**DOI:** 10.1186/s12864-020-6525-0

**Published:** 2020-01-31

**Authors:** Larry A. Cogburn, Nares Trakooljul, Xiaofei Wang, Laura E. Ellestad, Tom E. Porter

**Affiliations:** 1 Department of Animal and Food Sciences, University of Delaware, Newark, DE 19717 USA; 2 Leibniz Institute for Farm Animal Biology, Institute for Genome Biology, Wilhelm-Stahl-Allee 2, 18196 Dummerstorf, DE Germany; 3 Department of Biological Sciences, Tennessee State University, Nashville, TN 37209 USA; 4 Department of Poultry Science, University of Georgia, Athens, GA 30602 USA; 5 Department of Avian and Animal Sciences, University of Maryland, College Park, MD 20742 USA

**Keywords:** Up-stream regulators, Target genes, Lipid metabolism, Lipolysis, Lipogenesis, Thermogenesis, Gene interaction networks, *Homeorhesis*, Spot 14 (THRSPA), THRSP paralogs, Metabolic switch, *ying-yang* metabolic regulation, Reciprocal inhibition/activation

## Abstract

**Background:**

The fasting-refeeding perturbation has been used extensively to reveal specific genes and metabolic pathways that control energy metabolism in the chicken. Most global transcriptional scans of the fasting-refeeding response in liver have focused on juvenile chickens that were 1, 2 or 4 weeks old. The present study was aimed at the immediate post-hatch period, in which newly-hatched chicks were subjected to fasting for 4, 24 or 48 h, then refed for 4, 24 or 48 h, and compared with a fully-fed control group at each age (D1-D4).

**Results:**

Visual analysis of hepatic gene expression profiles using hierarchical and K-means clustering showed two distinct patterns, genes with higher expression during fasting and depressed expression upon refeeding and those with an opposing pattern of expression, which exhibit very low expression during fasting and more abundant expression with refeeding. Differentially-expressed genes (DEGs), identified from five prominent pair-wise contrasts of fed, fasted and refed conditions, were subjected to Ingenuity Pathway Analysis. This enabled mapping of analysis-ready (AR)-DEGs to canonical and metabolic pathways controlled by distinct gene interaction networks. The largest number of hepatic DEGs was identified by two contrasts: D2FED48h/D2FAST48h (968 genes) and D2FAST48h/D3REFED24h (1198 genes). The major genes acutely depressed by fasting and elevated upon refeeding included *ANGTPL, ATPCL, DIO2, FASN, ME1, SCD, PPARG, SREBP2* and *THRSPA—*a primary lipogenic transcription factor. In contrast, major lipolytic genes were up-regulated by fasting or down-regulated after refeeding, including *ALDOB, IL-15, LDHB, LPIN2, NFE2L2, NR3C1, NR0B1, PANK1, PPARA, SERTAD2* and *UPP2*.

**Conclusions:**

Transcriptional profiling of liver during fasting/re-feeding of newly-hatched chicks revealed several highly-expressed upstream regulators, which enable the metabolic switch from fasted (*lipolytic/gluconeogenic*) to fed or refed (*lipogenic/thermogenic*) states. This rapid *homeorhetic* shift of whole-body metabolism from a catabolic-fasting state to an anabolic-fed state appears precisely orchestrated by a small number of ligand-activated transcription factors that provide either a fasting-lipolytic state (*PPARA, NR3C1, NFE2L2, SERTAD2, FOX01, NR0B1, RXR)* or a fully-fed and refed lipogenic/thermogenic state (*THRSPA, SREBF2, PPARG, PPARD, JUN, ATF3, CTNNB1*). *THRSPA* has emerged as the key transcriptional regulator that drives lipogenesis and thermogenesis in hatchling chicks, as shown here in fed and re-fed states.

## Background

The first few days after hatching pose the most critical period in the chicken’s terrestrial life. Upon hatching, the chick must sharply increase its metabolic rate to achieve and maintain an exceptionally high core temperature (41–42 °C) for life. The hatchling chick emerges from the egg shell with a retracted yolk sac, a lipid-drenched gut, and a lipid-laden liver that ensures its survival for two or 3 days, even without feeding. After consuming its first meal, the hatchling chick launches a predominant lipogenic drive in its major metabolic organ—the liver.

We have developed functional genomic tools and genetic resources to gain a global view and more detailed understanding of genes and gene interaction networks that regulate important biological processes (e.g., growth, metabolism and development) in the chicken [1–4]. Using our original chicken (3.2 K) liver cDNA microarray, we explored time-course transcriptional profiles in liver of chickens during the embryo-to-hatching transition and high-growth (HG) and low-growth (LG) chickens during fasting and refeeding [1, 3, 4]. The transcriptional analysis of liver in late embryos and newly hatched chicks revealed two distinct gene expression patterns. Cluster “A” genes were highly expressed in late embryos (e16-e20) and depressed in hatchlings (d1-d9). In contrast, Cluster “B” genes were low in late embryos and sharply elevated after hatching. In a second study using the 3.2 K liver array, we examined transcriptional profiles in HG and LG chickens during an episode of fasting and refeeding at 6wk. Furthermore, we discovered several clusters of functionally-related hepatic genes that respond to the abrupt metabolic perturbation of the embryo-to-hatchling transition or fasting and refeeding. These clusters of differentially-expressed genes (DEGs) were composed of several transcription factors (*THRSPA, PPARA, PPARG,* and *SREBF1*), growth factors (*IGF1, ATRN*), metabolic enzymes (*FASN, SCD, ME* and *PCK1*) and transport proteins (*FABP1, IGFBP4*). Recently, we used the Affymetrix Chicken Genome Chip® to expand the repertoire of hepatic genes involved in the homeorhetic regulation of metabolism during the peri-hatch period [5]. Our study provided a higher resolution of transcriptional responses during the switch from ectothermic (embryo) to endothermic (hatchling) metabolism. We also confirmed and expanded the number DEGs that populate two distinct clusters of hepatic genes with opposing expression patterns, which we originally reported with our low-density 3.2 K liver array [1, 3]. Thus, *THRSPA* has emerged as the major transcription factor and highest-expressed hepatic gene supporting enhanced lipogenesis and thermogenesis in newly-hatched chicks [5]. The transcriptional choreography of the abrupt switch from lipolysis in late embryos to lipogenesis/thermogenesis in hatchling chicks appears to be controlled by about 30 microRNAs (miRNAs), which selectively target major hepatic transcription factors and their downstream metabolic genes [6]. Further, this RNA sequencing of liver has revealed reciprocal expression patterns of numerous miRNAs and their metabolic gene targets during the embryo-to-hatchling transition.

The fasting-refeeding perturbation has been used extensively to uncover specific genes and pathways that control energy metabolism in chickens of various ages. For example, the transcriptional analysis of liver in 4-wk-old broiler chickens fasted for either 16 or 48 h revealed four hierarchical clusters of functionally related genes, where the majority of metabolic DEGs were down-regulated by prolonged fasting [7]. Compared to the control (fed) group, hepatic genes controlling β-oxidation of fatty acids, gluconeogenesis and ketogenesis were up-regulated by fasting, while genes involved in fatty acid and cholesterol biosynthesis were highly expressed in fed birds, with the notable exception of HMG-CoA synthase 1 (*HMGCS1*), which was up-regulated by prolonged fasting of 4wk broiler chickens. Earlier, we used a chicken 20.7 K oligo microarray for transcriptome profiling of the hypothalamus in broiler chicks during an episode of fasting and refeeding immediately post-hatching, D0-D4 [8]. This transcriptional study of the hypothalamus demonstrates the importance of the neuropeptide Y and melanocortin pathways in regulation of metabolic and regulatory responses to fasting and refeeding in newly-hatched chicks. A subsequent microarray analysis of the hypothalamus in 2wk broiler chickens, which were fasted for 24 h or 48 h, or fasted for 48 h, then refed for 24 h [9], confirmed our original report of opposing actions of hypothalamic orexigenic and anorexigenic pathways in the switch from glucose metabolism in fed (and refed) chicks to lipid catabolism of chickens fasted for either 24 h or 48 h [8].

Our immediate interest in the present study was examination of global patterns of hepatic gene expression in newly-hatched cockerels during a fasting-refeeding perturbation, given during the first 4 days (D0-D4) of terrestrial life. The first few days after hatching and the associated shift from metabolism of stored yolk to metabolism of ingested feed are critical for normal growth of the chicks. Failure to adequately make this shift can result in failure of the chicks to thrive and grow. Additionally, the time from hatching to provision of feed can vary in the poultry industry, due to timing of the hatch and distance for transportation of hatchling chicks to the rearing houses. However, relatively little is known about the mechanisms controlling the metabolic switch from lipolytic/gluconeogenic to lipogenic/glycolytic metabolism associated with initial feeding of newly hatched chicks. Previous studies of gene expression in regulatory and metabolic tissues during a bout of fasting and refeeding in the chicken have been implemented after chickens were weeks old (1, 2, 4 or 6 wk), well after depletion of residual yolk lipids and after metabolic and regulatory pathways are well established. An exception was the study of transcriptional regulation of hepatic lipogenesis in unfed hatched broiler chicks versus seven-day-old chicks by microarray analysis coupled with targeted qRT-PCR analysis of liver from one-wk-old chicks, which were withheld from feed for the first 48 h after hatching [10]. Delayed feeding of these hatchling chicks depressed the up-regulation of key lipogenic transcription factors (*THRSPA, SREBF1, SREBF2* and *PPARG*) and metabolic enzymes (*SCD, ME1, FASN, ACACA* and *ACLY*), which were normally induced with initial feeding. A recent study investigating the impact of delayed feeding at hatch on gene expression patterns in liver and breast muscle revealed perturbations in developmental profiles of *PPARG* and *CHREBP*, which indicates a transitional delay in the switch from lipid to carbohydrate metabolism in these tissues [11].

A chicken 20.7 K oligo microarray was used to examine hepatic transcriptomes of fasting (4, 24 and 48 h), refeeding (4, 24 and 48 h) and fully-fed (D1-D4) broiler chicks using the same experimental design described previously for transcriptional analysis of the hypothalamus in newly-hatched chicks during fasting and refeeding [8]. Pairwise comparisons of 10 treatment groups allowed identification of hundreds of differentially expressed genes (DEGs), including major transcription factors and their direct down-stream targets, which control lipid metabolism via gene interaction networks that control metabolic and regulatory pathways during the first 4 days post-hatching (D0-D4). Our analysis has identified several major transcription factors and their coactivators and co-inhibitors that govern *ying-yang* regulation of the switch from *lipolytic* to *lipogenic* metabolism in liver of newly-hatched chicks.

## Results

### Physiological measurements

**Fig. 1:**
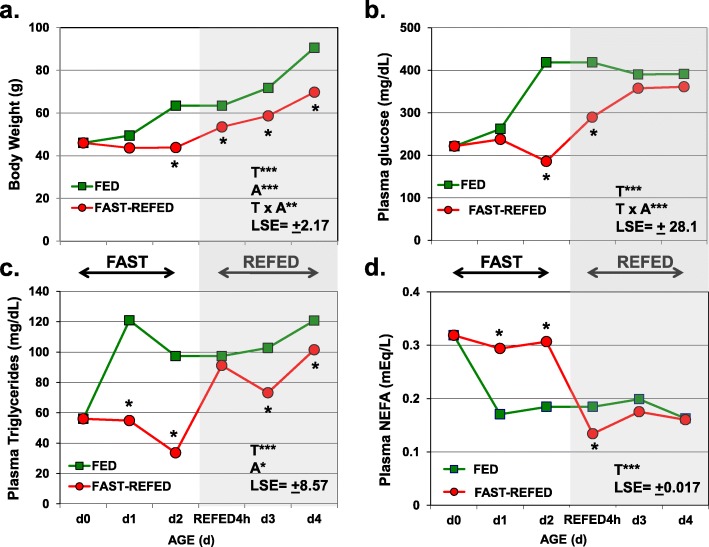
Body weight (**a**) and plasma metabolite [glucose (**b**), triglycerides (**c**) and (**d**) non-esterified fatty acids (NEFA)] responses of hatchling chicks. Each value represents the least square mean (LSM) and error (LSE) of five cockerels. The first three data points represent fasting treatment levels (D0FAST4h; D1FAST24h and D2FAST48h), while the last three data points (shaded area) represent refeeding treatment levels (D2REFED4h, D3REFED24h and D4REFED48h). The analysis of variance (ANOVA), using Type III error, indicates overall level of significance (*P ≤ 0.05; ***P ≤ 0.0001) for the main effects of fasting-refeeding treatments (T) and age (A), and their interaction (T x A) [shown in shaded area]. A single asterisk, below or above treatment points, indicates a significant difference (*P* ≤ 0.05) for each pairwise contrast between a fully-fed (FED) control group and a fasting-refeeding treatment. Note that the D2FED control group was used for both the D2FAST48h and D2REFED4h contrast

The metabolic response of newly-hatched (D0-D4) chicks to the fasting and re-feeding perturbation was evaluated by several physiological (phenotypic) measurements during after hatching, including body weight (Fig. 1a), plasma glucose (Fig. 1b), triglycerides (Fig. 1c) and non-esterified fatty acids (NEFA; Fig. 1d). Analysis of variance (ANOVA) showed significant (*P* ≤ 0.0001) main effects of treatment (T) and age (A) and the interaction of T x A (*P* ≤ 0.001) for body weight. At hatching (Day 0; D0), the average body weight (BW) was 46 g (Fig. 1a). On the last day of treatment (D4), the BW of the fully-fed group (D4FED96h or T9) was almost 2-fold higher than the D0FAST4h (T1) group at hatching. The BW of chicks fasted for 48 h (D2FAST48h or T5) was only 70% of the fully-fed group (D2FED48h or T4). And likewise, the BW of the chicks refed for 48 h (D4REFED48h or T10) was 23% lower than that of the fully-fed group on Day 4 (D4FED96h).

Plasma glucose levels showed a main effect of treatment and a T x A interaction (*P* ≤ 0.0001). Glycemia differed among treatment groups on Day 2, where fed chicks (D2FED48h) had higher (*P* ≤ 0.05) levels of circulating glucose than D2FAST48h or D2REFED4h (T6) chicks (Fig. 1b). Plasma glucose was similar for Day 3 and Day 4 treatment groups (375 mg/dL), which was within the normal glycemia range for the chicken. Plasma triglyceride levels (Fig. 1c) were dramatically depressed (*P* ≤ 0.05) in chicks fasted for 4, 24 or 48 h (an average level of 54.9 mg/dL) when compared to that (120.9 mg/dL) of fully-fed chicks (D1FED24h or T2; and D2FED48h or T4). Circulating triglycerides levels of refed groups (D3REFED24h or T8; and D4REFED48h or T10) were lower than their respective fully-fed control groups (D3FED72h or T7; and D4FED96h or T9). Plasma NEFA levels were elevated (*P* ≤ 0.05) in fasted chicks (D0FAST4h; D1FAST24h; and D2FAST48h) when compared to fed chicks (D1FED24h and D2FED48h) (Fig. 1c). Plasma NEFA levels of fasted chicks at 4 h after refeeding (D2REFED4h) were lower (*P* ≤ 0.05) than fully-fed chickens (D2FED24h) (Fig. 1d). However, plasma NEFA levels in the D3REFED24h and D4REFED48h chicks were similar to their respective control fed groups (D3FED72h; and D4FED96h).

### Preliminary visual analysis of DEGs using GeneSpring GX software

#### Preliminary Venn diagram

First, we used GeneSpring GX software with default settings to determine the number of DEGs for three inclusive treatment groups: FAST (4, 24, 48 h), FED (D1-D4) and REFED (4, 24, 48 h). A Venn diagram (not shown) revealed the distribution of these DEGs [FAST (1459 DEGs)), FED (243 DEGs) and REFED (1658 DEGs)], the number of unique FAST (608 DEGs), FED (54 DEGs) and REFED (794 DEGs) and the number of commonly-shared genes (130 DEGs) and the number shared between FAST and REFED (698 DEGs). Clearly, the three FAST (6-fold higher) and REFED (6.8-fold higher) conditions provoked a greater number of DEGs than did the four FED conditions.

#### Unsupervised hierarchical clustering and heat map

**Fig. 2:**
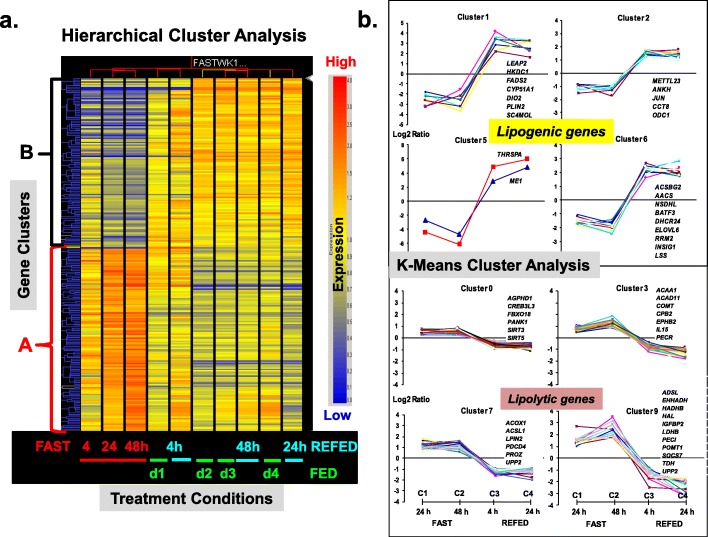
Initial hierarchical clustering analysis of differentially-expressed genes (DEGs) (*P* ≤ 0.05) identified in liver of newly-hatchling chicks during the fasting-refeeding perturbation (Panel **a**). This heat map, representing two-way hierarchical clustering of 1170 DEGs (Y-axis) across 10 treatment groups (X-axis), shows two major clusters of DE genes that are either up-regulated (Cluster A) or down-regulated (Cluster B) by fasting (4, 24 and 48 h) after hatch. In contrast, Cluster A genes are down-regulated in the fully-fed (FED) and refed (REFED) groups on day 3(D3) and D4, whereas Cluster B genes are up-regulated after refeeding and in FED groups on D2, D3 and D4**.** Panel **b**. K-means cluster plots of DEGs (log2 FC) identified in four contrasts of fasting [C1 = D1FED vs. D1FAST24h; and C2 = D2FED vs. D2FAST48h) and refeeding (C3 = D2FAST48h vs. D3REFED4h; and C4 = D2FAST48 vs. D3REFED24h). K-means analysis revealed two distinct gene expression patterns, each composed of four clusters of DEGs identified by microarray and statistical analysis**.** Four distinct K-means clusters of lipogenic genes were down-regulated by fasting and sharply rebounded at 4 h or 24 h after refeeding. In contrast, four other clusters represent lipolytic genes whose expression was up-regulated by fasting and sharply down-regulated after refeeding. The original responses showed positive or negative log2 FC values which represent down-regulation or up-regulation by fasting, respectively. Further, positive or negative log2 FC means indicates either down-regulation or up-regulation caused by re-feeding for either 4 h or 24 h after a 48 h fast, respectively. However, the log2 FC values shown here were multiplied by − 1 to make the relative expression (log2 fold-change) either positive for up-regulation or negative for down-regulation of gene expression. Several examples of major metabolic DEGs are provided for each cluster. An annotated list of DEGs identified in each K-means cluster is provided in Additional file 1, which also includes a composite graph of all K-means clusters including the low-amplitude changes in Clusters 4 and 8, both of which were down-regulated with fasting and sharply up-regulated with refeeding

The heat map (Fig. 2a) generated by GeneSpring software illustrates hierarchical clustering of 958 DEGs (FDR adjusted *P* ≤ 0.05) genes (Y-axis) across 10 treatment groups (X-axis). Two major clusters of DEGs were identified, where Cluster A represents about 52% of all DEGs, which were sharply upregulated in all three fasted groups (D0FAST4h, D1FAST24h and D2FAST48h) and down-regulated in both the fully-fed groups (D3FED48h and D4FED96h) and the re-fed groups (D3REFED24h and D4REFED48h). In contrast, the expression of the other half of all DEGs was down-regulated by prolonged fasting for either 24 or 48 h (Cluster B). With the exception of the D0FAST4h and D1FED24h groups, treatment clusters show that the expression of genes in Cluster A was down-regulated in fully-fed groups (D2FED, D3FED and D4FED) and refed groups (D3REFED24h and D4REFED48h). Likewise, the DEGs in Cluster B were highly expressed in fed and refed groups on D3 and D4 of treatment. The majority of DEGs in the D1FED and D2REFED4h treatment groups were also highly expressed and clustered together in the condition (treatment) tree. This dataset from unsupervised hierarchical clustering was only used for preliminary visual analysis of gene expression patterns.

#### K-means cluster analysis

A more stringent statistical analysis, afforded by K-means clustering, was used to identify clusters of functionally-related DEGs. Four pairwise contrasts were used to visually identify DEGs that responded to fasting (D1FAST24h and D2FAST48h) or refeeding (D2REFED4h and D3REFED24h). The K-means cluster analysis identified 196 DEGs (FDR adjusted *P* ≤ 0.05), which form 10 clusters (Cluster 0–9) of functionally-related DEGs (Additional file 1). Plots of eight K-means clusters (Fig. 2b) provide a detailed view of two distinct gene expression patterns, while two additional K-means clusters of lipogenic genes with low amplitude responses were not presented in this figure. Four clusters had low expression during fasting (24 h or 48 h), and a sharp log-scale increase in abundance was found at 4 h or 24 h after refeeding (e.g., *THRSPA, ME1, SCD, DIO2,* and *PLIN2*). In contrast, fasting for 24 h or 48 h increased expression of four gene clusters, while refeeding (4 h or 24 h) reduced hepatic expression, albeit at lower amplitude (e.g., *ACAA1, IGFBP2, LPIN2, SIRT5* and *UPP2)*. The genes found in Clusters 1, 2, 5 and 6 (Fig. 2b) were down-regulated by fasting and upregulated by refeeding after a 48 h fast. These lipogenic genes are involved in energy metabolism and synthesis of fatty acids. For example, Cluster 5 represents the two most abundant DEGs: thyroid hormone responsive Spot 14 protein alpha (*THRSPA*), a major lipogenic transcription factor, and malic enzyme (*ME1*), a key enzyme controlling fat biosynthesis; expression of both genes was depressed by fasting and sharply rebound after refeeding. Three additional clusters of lipogenic DEGs (Clusters 1, 2 and 6) have identical expression patterns, with highest expression in the refed state. In contrast, the lipolytic DEGs found in Clusters 0, 3, 7, and 9 were upregulated by fasting and down-regulated upon refeeding. Many of these genes are involved in fat catabolism and acute phase responses (*ACAA1, HMGCL, HMGCS1, LDHB, LPIN2, SIRT3* and *SIRT5*). Annotated lists and plots of DEGs assigned to all 10 K-means clusters are provided in Additional file 1.

### Comparison of DEGs identified by microarray analysis vs. “analysis ready” (AR)-DEGs used for ingenuity pathway analysis (IPA)

The microarray DEGs that mapped to known mammalian genes accrued in the Ingenuity Knowledge Base were considered as AR-DEGs by IPA. However, almost one-quarter of the chicken-specific DEGs determined from our microarray analysis were rejected by IPA due to the absence of a valid Entrez Gene ID accrued in the mammalian-centric Ingenuity Knowledge Base (Additional file 2). Chicken transcripts possessing a genomic locus prefix (LOC) ID number or an avian-specific gene ID were largely rejected by IPA. This difference between microarray DEGs and the reduced number of AR-DEGs accepted by IPA for functional analysis of chicken genes could also be attributed to a large number of un-annotated oligo probes (23%) found on the chicken 20.7 K oligo array, which was last annotated in 2009 [12].

**Fig. 3:**
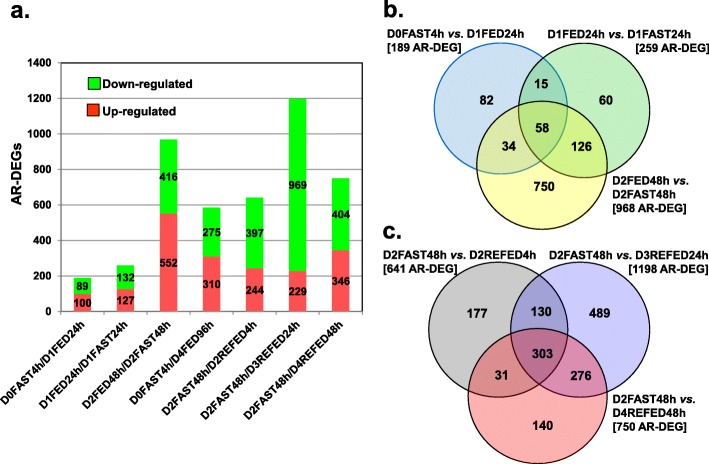
Stacked-bar graph of seven pairwise treatment contrasts showing the highest numbers of up-regulated and down-regulated “Analysis Ready” (AR)-DEGs among the 45 possible pairwise contrasts of 10 treatment conditions (Panel **a**). The Venn diagrams provide the numbers of unique and commonly shared AR-DEGs found within the meaningful contrasts. The Venn diagram in Panel **b** compared three contrasts of AR-DEGs in chicks that were either fasted for 4 h immediately after hatching (D0FAST4h), fasted for 24 h (D1FAST24h) or fasted for 48 h (D2FAST48h) versus chicks that were either fully-fed for 24 h (D1FED24h) or 48 h (D2FED48h). Likewise, the recovery from prolonged fasting (D2FAST48h) was examined by three refeeding contrasts (D2REFED4h, D3REFED24h or D4REFED48h) (Panel **c**). The number of AR-DEGs found in each contrast is shown in brackets, while numbers within arcs represents genes shared between or among contrasts. Annotated lists of AR-DEGs found in the five contrasts are provided by multiple worksheets in Additional file 3

The numbers of up-regulated and down-regulated AR-DEGs for seven contrasts are presented in a stacked bar graph (Fig. 3a). Fewer AR-DEGs were found in the D0FAST4h vs. D1FED24h contrast (189 AR-DEGs) and D1FED24h vs. D1FAST24h contrast (259 AR-DEGs). The greatest number of AR-DEGs was found in the D2FED48h vs. D2FAST48h (968 AR-DEGs) and D2 FAST48h vs. D3REFED24h (1198 AR-DEGs) contrasts. The Venn diagram in Fig. 3b shows the three-way comparison of D0FAST4h vs. D1FED24h, D1FED24h vs. D1FAST24h, and D2FED48h vs. D2FAST48h contrasts. The largest number of unique DEGs (750 AR-DEGs) was found in the D2FED48h vs. D2FAST48h, while only 58 AR-DEGs were commonly shared among the three contrasts. The second Venn diagram (Fig. 3c) compared the number of AR-DEGs found in three fasting-refeeding contrasts: D2FAST48h vs. D2REFED4h, D3REFED24h or D4REFED48h. The largest number of unique genes (489 AR-DEGs) was found in the D2FAST48h vs. D2REFED24h, while 303 AR-DEGs were commonly-shared among all three fasting-refeeding contrasts. Annotated lists of the AR-DEGs from five meaningful contrasts, which were used for IPA, are presented in Additional file 3.

### IPA of five prominent pairwise contrasts during a fasting-refeeding perturbation (D1-D4)

#### Effects of 24 h fasting (D1FED24h vs. D1FAST24h contrast)

A summary of IPA of liver transcriptomes from the D1FED24h vs. D1FAST24h contrast is presented in Table 1. The top canonical pathways overpopulated by AR-DEGs from this contrast were related to tryptophan degradation and biosynthesis of cholesterol and nicotinamide adenine dinucleotide (NAD). The top three upstream regulators identified by IPA in the D1FED24h vs. D1FAST24h contrast were *SREBF2, PPARA* and *SREBF1.* The most highly represented subcategories under the IPA “Molecular and Cellular Functions” category were “Small Molecule Biochemistry” (94 AR-DEGs), “Lipid Metabolism” (66 AR-DEGs) and “Molecular Transport” (68 AR-DEGs). The “Physiological System Development and Function’ category in IPA contained the subcategories: “Connective Tissue Development and Function” (26 AR-DEGs), “Digestive System Development and Function” (17 AR-DEGs), “Hepatic System Development and Function” (17 AR-DEGs), “Organ Morphology” (21 AR-DEGs) and “Organismal Development (19 AR-DEGs)”. Among the most highly expressed up-regulated genes in the D1FED24h treatment were *THRSPA*, deiodinase 2 (*DIO2*), *ME1* and *PLIN2.* The top down-regulated genes (negative log2 ratios) in the D1FAST24h liver were uridine phosphorylase 2 (*UPP2*), cytochrome P450 family 4 subfamily A member 22 (*CYP4A22*), 3-hydroxy-3-methylglutaryl-CoA synthase 1 (*HMGCS*1) and cytochrome P450, family 2, subfamily c, polypeptide 44 (*CYP2C44*).

**Table 1:** IPA summary of liver transcriptomes in-hatchling chicks--D1FED24h vs. D1FAST24h contrast

Top Canonical Pathways	*p*-value	Overlap	Ratio
Tryptophan Degradation (Eukaryotic)	1.15E-12	42.9%	9/21
Super-pathway of Cholesterol Biosynthesis	9.65E-10	28.6%	8/28
Cholesterol Biosynthesis I-III	4.48E-09	46.2%	6/13
NAD Biosynthesis II	9.23E-09	40.0%	6/15
Tryptophan Degradation 2-amino-3-carboxymuconate	9.83E-09	62.5%	5/8
Top Upstream Regulators	*p*-value of overlap	# Target genes	
* SREBF2*	1.08E-15	19	
* PPARA*	9.38E-14	27	
* SREBF1*	2.55E-13	15	
* PPARG*	3.15E-09	24	
* PPARGC1A*	1.07E-08	18	
Top Molecular and Cellular Functions	*p*-value	# Genes	
Amino Acid Metabolism	9.80E-03 - 6.26E-11	25	
Small Molecule Biochemistry	1.19–02 - 6.26E-11	94	
Lipid Metabolism	1.19–02 - 2.16E-08	66	
Molecular Transport	1.19–02 - 2.16E-08	68	
Vitamin and Mineral Metabolism	5.45E-03 - 7.14E-07	21	
Physiological System Development and Function	*p*-value	# Genes	
Connective Tissue Development and Function	1.13E-02 - 3.88E-04	26	
Digestive System Development and Function	1.02E-02 - 4.29E-04	17	
Hepatic System Development and Function	6.03E-03 - 4.29E-04	17	
Organ Morphology	1.13E-02 - 4.29E-04	21	
Organismal Development	1.13E-02 - 4.29E-04	19	
Top Up-regulated genes	D1FED24h/D1FAST24h	Top Down-regulated genes	D1FED24h/D1FAST24h
* THRSPA*	4.46	*UPP2*	−2.70
* DIO2*	3.24	*CYP4A22*	−2.01
* HKDC1*	3.22	*HMGCS1*	−1.89
* ME1*	2.75	*CYP3A7*	−1.73
* PLIN2*	2.63	*TDH*	−1.62
* FADS2*	2.47	*FKBP5*	−1.62
* MSMO1*	2.21	*ADSL*	−1.60
* CYP51A1*	2.06	*ECI2*	−1.52
* SCD*	1.74	*CYP2C44*	−1.44
* BATF3*	1.72	*LDHB*	−1.37

Ingenuity Pathway Analysis (IPA) was used for functional analysis of 259 DEGs (FDR adj. *P* ≤ 0.05) that were also “Analysis Ready” (AR)-DEGs from the D1FED24h vs. D1FAST24h contrast. The top 10 up-regulated and down-regulated AR-DEGs are presented along with their respective log2 ratio of treatment conditions

**Fig. 4:**
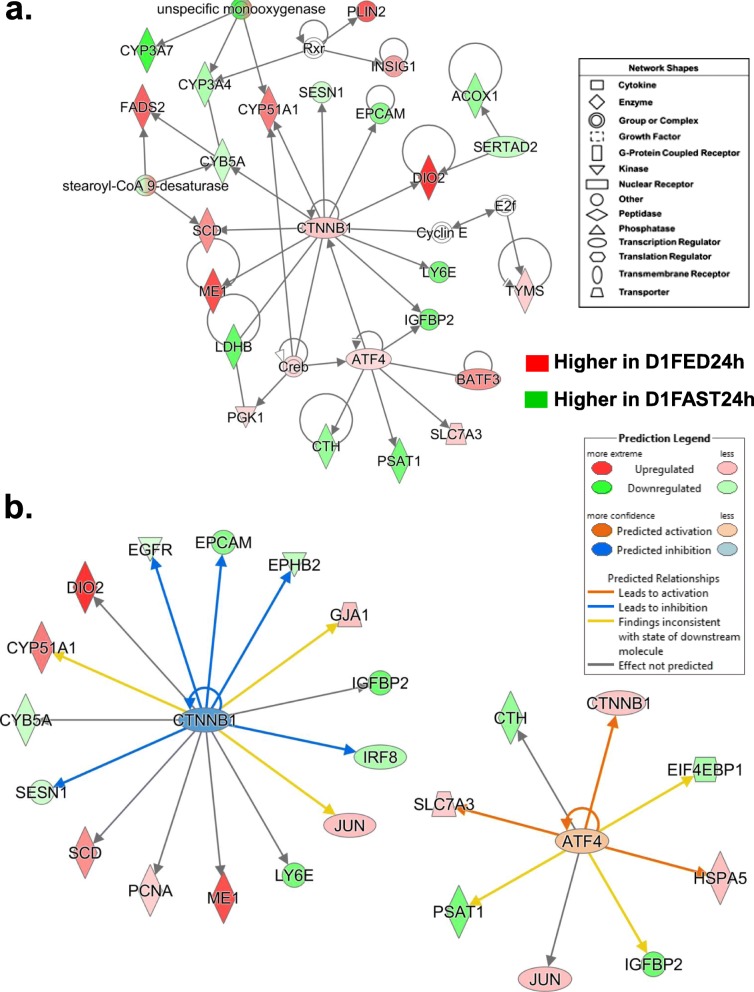
A gene interaction network (Panel **a**) of lipogenic (green symbols) and lipolytic (red symbols) AR-DEGS found in the D1FED24h vs. D1FAST24h contrast. This gene network was functionally annotated by IPA as “Lipid Metabolism/Molecular Transport”. These genes are differentially regulated by two transcription factors [catenin beta 1 (*CTNNB1*) and activating transcription factor 4 (*ATF4*)]. Ingenuity® Upstream Regulator Analysis identified additional direct targets for each transcription factor (Panel **b**) and predicts that *CTNNB1* should be inhibited due to down-regulation of its eight direct target genes (*EGFR, EPCAM, EPHB2, IGFBP2, IRF8, LY6E, SESN1* and *CYB5A*), although the expression of *CTNNB1* and seven direct target genes were up-regulated (i.e., higher in D1FED24h). Ingenuity correctly predicted activation of *ATF4* and up-regulation of four direct target genes including CTNNB1 and another transcription factor, Jun proto-oncogene, AP-1 transcription factor subunit (*JUN*)

The gene network depicted in Fig. 4a was centered on the interaction of three transcription factors: catenin beta 1 (*CTNNB1*), activating transcription factor 4 [*ATF4*; or cAMP-response element binding protein 2 (*CREB2*)] and SERTA domain containing 2 (*SERTAD2*), which is a newly discovered co-regulator of lipolysis, thermogenesis and oxidative metabolism. *SERTAD2* has a direct action on both *DIO2* and acyl-CoA oxidase 1 (*ACOX1*), which is the initial enzyme in the fatty acid β-oxidation pathway. This network was functionally annotated by IPA as “Lipid Metabolism” and “Molecular Transport”. Several lipogenic genes in this network are highly expressed in liver of the D1FED24h chicks, including *ME1*, *SCD, FADS2, CYP51A1*, *PLIN2, INSIG1, DIO2* and basic leucine zipper ATF-like transcription factor 3 (*BATF3*), a transcriptional repressor of the *JUN* oncogene. The cationic amino acid transporter (*SLC7A3*), a known target of *ATF4*, was slightly upregulated in the D1FED24h chicks as well as phosphoglycerate kinase 1 (*PGK1)*, a glycolytic enzyme and direct target of *CREB*. Several additional genes were expressed higher in the D1FAST24h treatment, including *PSAT1, CTH, LDHB, IGFBP2, LY6E, EPCAM, SESN1* and three monoxygenases (*CYB5A, CYP3A4 and CYP3A7*). The Ingenuity Upstream Regulator Analysis identified 15 direct targets of *CTNNB1* (Fig. 4b), 7 up-regulated and 8 down-regulated AR-DEGs. Ingenuity predicts that *CTNNB1* should be inhibited (blue symbol), which would lead to inhibition (blue arrows) of five direct target genes [epidermal growth factor receptor (*EGFR*), epithelial cell adhesion molecule (*EPCAM*), EPH receptor B2 (*EPHB2*) and sestrin 1 (SESN1)]. Ingenuity also predicts that ATF4 would be activated, which would lead to the activation of three direct target genes (*CTNNB1, HSPA5* and *SLC7A3*) as indicated by the orange-colored arrows.

#### Effects of 48 h fasting [D2FED48h vs. D2FAST48h contrast]

The summary of the Ingenuity Pathway Analysis of liver transcriptomes from the D2FED48h vs. D2FAST48h contrast is presented in Table 2. The major canonical pathways populated by AR-DEGs in this contrast were related to cholesterol biosynthesis, protein ubiquitination, tryptophan degradation and the oxidative stress response. The top transcription factors found in this contrast were hepatocyte nuclear factor 4 alpha (*HNF4A)*, peroxisome proliferator activated receptor alpha (*PPARA*), tumor protein p53 (*TP53*), nuclear factor, erythroid 2 like 2 (*NFE2L2*) and MYC proto-oncogene, bHLH transcription factor (*MYC*). The top IPA “Molecular and Cellular Functions” were “Lipid Metabolism” (195 AR-DEGs), “Small Molecule Biochemistry” (277 AR-DEGs) and “Cell Cycle” (175 AR-DEGs). Tissue morphology, tissue (connective) development, and organismal survival and development were the most populated subcategories in “Physiological System Development and Function” category of IPA. The top four up-regulated AR-DEGs in the D2FED48h vs. D2FAST48h contrast were *THRSPA, ME1, SCD*, and *FADS2*, while the highest expressed AR-DEGs in the D2FAST48h treatment were *IGFBP2,* adenylosuccinate lyase (*ADSL*)*, CYP3A7,* and *CYP4A22*.

**Table 2:** IPA summary of liver transcriptomes in hatchling chicks-D2FED48h vs. D2FAST48h contrast

Top Canonical Pathways	*p*-value	Overlap	Ratio
Superpathway of Cholesterol Biosynthesis	1.36E-09	42.9%	12/28
Protein Ubiquitination Pathway	2.83E-08	13.3%	34/255
Tryptophan Degradation III (Eukaryotic)	1.65E-07	42.9%	9/21
NRF2-mediated Oxidative Stress Response	2.46E-07	14.4%	26/180
Cholesterol Biosynthesis I	5.92E-07	53.8%	7/13
Top Upstream Regulators	***p*-value of overlap**	**# Target genes**	
HNF4A	1.32E-24	174	
PPARA	1.48E-24	69	
TP53	2.81E-19	119	
NFE2L2	3.18E-15	51	
MYC	1.73E-14	95	
Top Molecular and Cellular Functions	***p*-value**	**# Genes**	
Lipid Metabolism	1.83E-03 - 3.91E-15	195	
Small Molecule Biochemistry	2.08E-03 - 3.91E-15	277	
Cell Cycle	2.07E-03 - 1.26E-11	175	
Amino Acid Metabolism	2.08E-03 - 4.78E-11	41	
Cell Death and Survival	1.13E-03 - 5.19E-11	31	
Physiological System Development and Function	***p*-value**	**# Genes**	
Tissue Morphology	2.00E-03 - 4.53E-06	92	
Tissue Development	1.77E-03 - 9.24E-06	76	
Connective Tissue Development and Function	2.00E-03 - 1.09E-05	116	
Organismal Survival	2.13E-05 - 2.13E-05	200	
Organismal Development	1.77E-03 - 4.61E-05	28	
Top up-regulated genes	**D2FED48h/D2FAST48h**	**Top down-regulated genes**	**D2FED48h/D2FAST48h**
*THRSPA*	6.19	*IGFBP2*	−3.45
*ME1*	4.74	*ADSL*	−3.18
*SCD*	4.74	*CYP3A7*	−3.07
*FADS2*	3.63	*CYP4A22*	−2.74
*LGALS2*	3.47	*CYP2C44*	−2.66
*CYP2C44*	3.24	*LDHB*	−2.62
*PLIN2*	3.23	*UPP2*	−2.48
*MSMO1*	2.49	*EHHADH*	−2.41
*ELOVL6*	2.43	*HADHB*	−2.31
*INSIG1*	2.33	*BHMT*	−2.15

Ingenuity Pathway Analysis (IPA) was used for functional analysis of 968 Analysis Ready (AR)-DEGs from the D2FED48h vs. D2FAST48h contrast. The top 10 up-regulated and down-regulated AR-DEGs are presented along with their respective log2 ratio of treatment conditions

Additional file 4 provides annotated lists of AR-DEGs assigned by IPA to canonical pathways and biological processes, which were over-represented by the D2FED48h vs. D2FAST48h contrast (see Table 2). For example, IPA recognized 36 AR-DEGs that were involved in “Disorders of Lipid Metabolism”, where 14 AR-DEGs were more highly expressed in the D2FED48h group and 22 AR-DEGs had greater expression in the D2FAST48h chicks. The importance of “Oxidation of Fatty Acids” was confirmed by the greater abundance of 24 AR-DEGs in D2FAST48h chicks, whereas only 4 AR-DEGs were higher in the D2FED48h treatment. Likewise, the IPA canonical pathway “LPS/IL1 Inhibition of RXR Function” had 18 AR-DEGs that were more abundant in the D2FAST48h group, compared to only 8 genes with higher expression in the D2FED48h chicks. Another canonical pathway in IPA “NRF2-Mediated Oxidative Stress” was represented by 12 up-regulated and 14 down-regulated AR-DEGs from this contrast. Under the “LXR/RXR Activation” pathway, only 6 AR-DEGs were up-regulated, while 12 AR-DEGs were more abundant in the D2FAST48h treatment. Similarly, the “FXR/RXR Activation” pathway was recognized by 4 up-regulated and 10 down-regulated AR-DEGs. The “Coagulation System” was more active in the D2FAST48h treatment (7 AR-DEGs) than in the D2FED48h condition (1 AR-DEG).

**Fig. 5:**
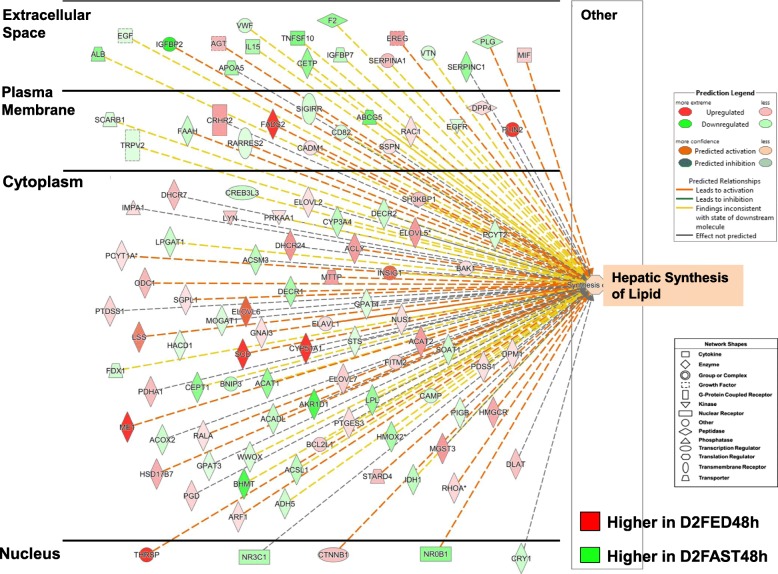
Subcellular distribution of 107 AR-DEGs functionally annotated by IPA as “Concentration of Lipids” from the D2FED48h vs. D2FAST48h contrast. This figure provides an overview of the transcriptional hierarchy of hepatic genes that control the concentration of lipids. Genes with red symbols are expressed higher in liver of D2FED48h cockerels, while green symbols indicate higher hepatic expression in the D2FAST48h treatment. A group of five upstream regulators control transcription of numerous metabolic enzymes, transporters, kinases and phosphatases in the cytoplasm, several transmembrane receptors, G-protein-coupled receptors, peptidases and enzymes in the plasma membrane, and even fewer growth factors, transporters and enzymes found in extracellular space. IPA predicts that the concentration of lipid in liver would be inhibited by the eight up-regulated transcription factors as indicated by the dashed blue lines, while yellow dashed lines represent inconsistence between the expected state and observed state of downstream genes. An annotated list of these 107 AR-DEGs controlling concentration of lipid is provided in Additional file 4

The subcellular distribution of 107 AR-DEGs that regulate “Synthesis of Lipid” in liver of chicks from the D2FED48h vs. D2FAST48h contrast is presented in Fig. 5. In this overview of lipid synthesis, AR-DEGs with red symbols are *lipogenic* genes, which are highly expressed in liver of D2FED48h chicks, whereas green symbols indicate greater abundance of *lipolytic* genes in the D2FAST48h treatment (Additional file 4). This subcellular distribution of 54 up-regulated and 53 down-regulated AR-DEGs clearly illustrates transcription control of lipid synthesis by five transcription factors (*THRSPA, NR3C1, CTNNB1, NROB1* and *CRY1*), which control expression of numerous downstream target genes. Most AR-DEGs were found in the cytoplasm (70 AR-DEGs, which are mainly metabolic enzymes, transporters, kinases and phosphatases) with fewer genes in the plasma membrane (15 AR-DEGs; receptors, transporters, enzymes, a peptidase and a kinase) and extracellular space (17 AR-DEGs; coagulation factors, cytokines, transporters and an enzyme).

#### Effects of 4 h re-feeding [D2FAST48h vs. D2REFED4h contrast]

The IPA summary of liver transcriptomes in the D2FAST48h vs. D2REFED4h contrast is presented in Table 3. The top canonical pathways represented in this contrast were related to cholesterol and zymosterol biosynthesis, protein ubiquitination and tryptophan degradation. The transcription factors and their respective direct target genes identified by IPA were HNF4A, PPARA, NFE2L2, TP53 and E2F transcription factor 1 (E2F1). “Cell Death and Survival”, “Lipid Metabolism” and “Small Molecular Biochemistry” were among the most represented “Molecular and Cellular Functions” found in this contrast. The largest number of AR-DEGs assigned to the “Physiological System Development and Function” category of IPA was associated with connective tissue and morphology of tissue. Among the highest expressed genes in the D2FAST48h treatment were *CYP4A22, UPP2, IGFBP2*, and N-myc downstream regulated 1 (*NDRG1*), whereas *THRSPA*, hexokinase domain containing 1 (*HKDC*), cytochrome P450 family 51 subfamily A member 1 (*CYP51A1*) and methylsterol monooxygenase 1 (*MSMO1*) were the most abundant AR-DEGs found in the D2REFED4h treatment.

**Table 3:** IPA summary of liver transcriptomes in hatchling chicks–D2FAST48h vs. D2REFED4h contrast

Top Canonical Pathways	*p*-value	Overlap	Ratio
Super-pathway of Cholesterol Biosynthesis	4.68E-09	35.7%	10/28 Cholesterol
Biosynthesis I-III	3.23E-08	53.8%	7/13
Protein Ubiquitination Pathway	9.27E-07	9.4%	24/255
Tryptophan Degradation III	5.12E-06	28.0%	7/25
Zymosterol Biosynthesis	1.01E-05	66.7%	4/6
Top Upstream Regulators	***p*-value of overlap**	**# Target genes**	
HNF4A	1.64E-16	126	
PPARA	3.12E-15	46	
NFE2L2	2.26E-13	44	
TP53	3.37E-13	92	
E2F1	2.26E-11	44	
Top Molecular and Cellular Functions	***p*-value**	**# Genes**	
Cell Death and Survival	7.95E-03 - 5.11E-10	206	
Lipid Metabolism	7.07E-03 - 6.42E-09	118	
Small Molecule Biochemistry	7.12E-03 - 6.42E-09	160	
Vitamin and Mineral Metabolism	7.07E-03 - 4.29E-08	39	
Cellular Growth and Proliferation	8.01E-03 - 7.35E-08	217	
Physiological System Development and Function	***p*-value**	**# Genes**	
Reproductive System Development and Function	4.46E-05 - 4.46E-05	8	
Connective Tissue Development and Function	7.76E-03 - 6.39E-05	89	
Embryonic Development	5.24E-03 - 1.23E-04	29	
Cardiovascular System Development and Function	5.24E-03 - 1.49E-04	10	
Tissue Morphology	6.24E-03 - 1.99E-04	63	
Top Up-regulated genes	**FAST48h/REFED4h**	**Top Down-regulated genes**	**FAST48h/REFED4h**
*CYP4A22*	2.53	*THRSPA*	−4.89
*UPP2*	2.49	*HKDC1*	−4.17
*IGFBP2*	1.79	*CYP51A1*	−3.60
*NDRG1*	1.77	*MSMO1*	−3.44
*TDO2*	1.68	*PLIN2*	−3.37
*VSNL1*	1.68	*ME1*	−2.78
*HAL*	1.56	*UGP2*	−2.72
*TDH*	1.53	*AACS*	−2.68
*CEPT1*	1.51	*ACSBG2*	−2.50
*SLC16A5*	1.46	*INSIG1*	−2.43

Ingenuity Pathway Analysis (IPA) was used for functional analysis of 641 “Analysis Ready” (AR)-DEGs found in the D2FAST48h vs.D2REFED4h contrast. The top 10 up-regulated and down-regulated AR-DEGs are presented along with their respective log2 ratio of treatment conditions

**Fig. 6:**
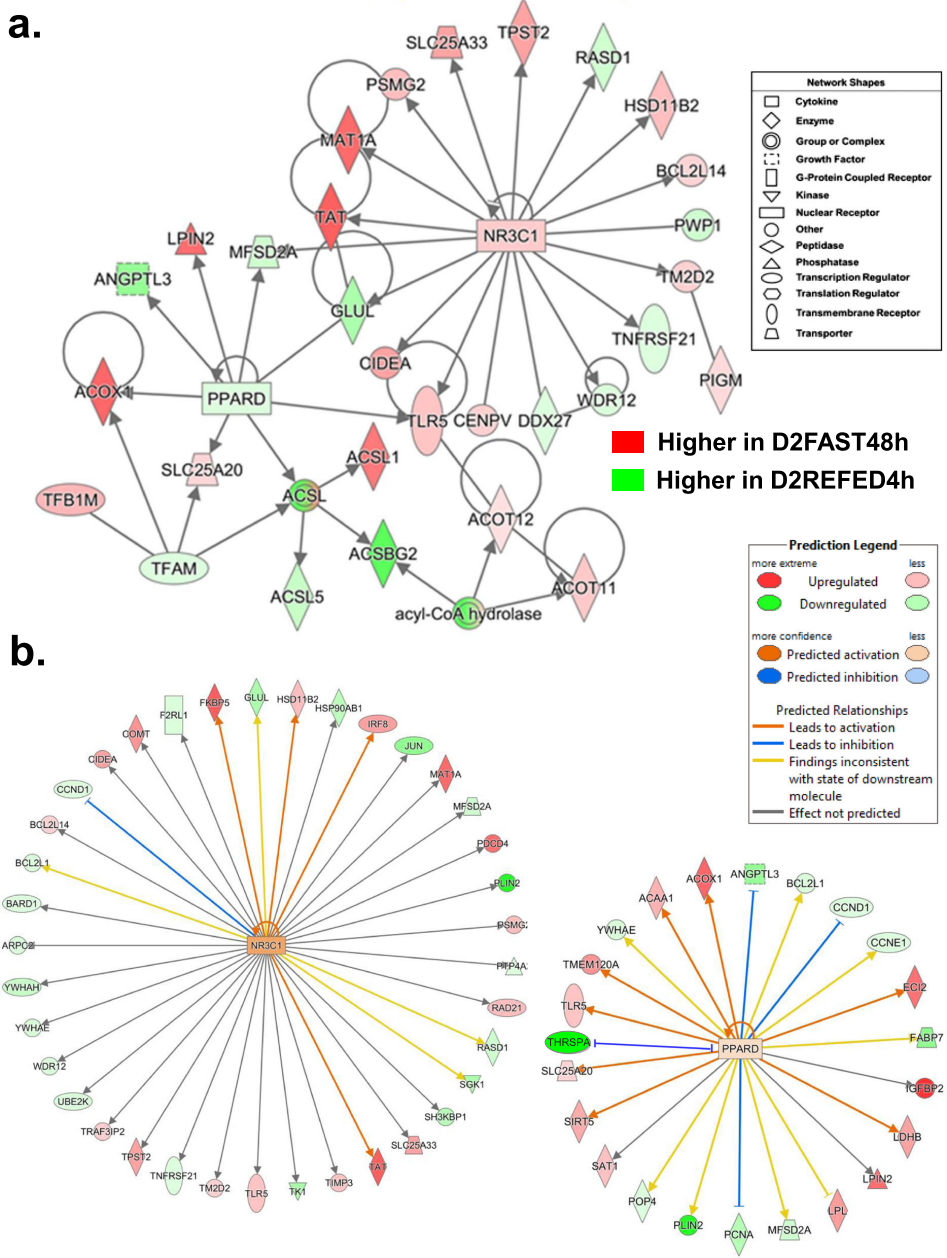
This gene network, identified in the D2FAST48h vs. D2REFED4h contrast, shows interactions between two transcription factors [nuclear receptor subfamily 3 group C member 1 (*NR3C1*) and peroxisome proliferator activated receptor delta (*PPARD*)] and their respective direct target genes (Panel **a**). This network was functionally annotated by IPA as “Endocrine Function” and “Lipid Metabolism”. Ingenuity Upstream Analysis predicts activation of NR3C1 (glucocorticoid receptor, GCR; Panel **b**), based on 18 up-regulated and 20 down-regulated AR-DEGs and slightly activated *PPARD,* based on the up-regulated state of 12 direct targets (red gene symbols) compared to 11 down-regulated genes (green gene symbols). Actually, the green gene symbols indicate higher expression in liver of D2REFED4h cockerels, while red gene symbols indicate higher expression in the D2FAST48h treatment group

The gene interaction network shown in Fig. 6a was functionally annotated by IPA as “Lipid Metabolism” and “Endocrine Function”. This network is centered on the interactions of two transcription factors *NR3C1* (or GCR) and peroxisome proliferator activated receptor delta (*PPARD*) with their direct target genes, three of which are shared [major facilitator superfamily domain containing 2A (*MFSD2A*), glutamate-ammonia ligase (*GLUL*) and toll like receptor 5 (*TLR5*)]. Methionine adenosyltransferase 1A (*MAT1A*) and tyrosine aminotransferase (TAT) were the most abundant direct targets of *NR3C1*, and were highly expressed in the D2FAST48h treatment*.* Although *PPARD* and four of its target genes, including angiopoietin like 3 (*ANGPTL3*) and acyl-CoA synthetase long chain family member 5 (*ACSL5*), were expressed more highly in the D2REFED4h treatment, four other targets of PPARD [*LPIN2,* acyl-CoA oxidase 1 (*ACOX1*)*, SLC25A20* and *ACSL1*] were more highly expressed in liver of the D2FAST48h chicks. Ingenuity Upstream Regulator Analysis identified 38 AR-DEGs in this contrast as direct targets of NR3C1 (Fig. 6b), 18 AR-DEGs were up-regulated and 20 AR-DEGs were down-regulated in this contrast. Ingenuity predicted that *NR3C1* was activated (orange symbol), which would lead to activation of four target AR-DEGs [FK506 binding protein 5 (*FKBP5*), hydroxysteroid 11-beta dehydrogenase 1 (*HSD11B1*), interferon regulatory factor 8 (*IRF8*) and *TAT*] as shown by the orange arrows. Ingenuity also identified 24 AR-DEGs as direct targets of *PPARD*, which was predicted to be slightly activated (orange symbol) since 8 of its direct targets were up-regulated in this contrast as indicated by the orange arrows. Ingenuity predicted inhibition of four target AR-DEGs (*ANGPTL3, CCND1, PCNA* and *THRSPA*) as depicted by the blunted blue lines.

#### Effects of 24 h re-feeding [D2FAST48h vs. D3REFED24h contrast]

The IPA summary of liver transcriptomes in the D2FAST48h vs. D3REFED24h contrast is presented in Table 4. The major canonical pathways identified in this contrast were related to tryptophan degradation, oxidative stress response, cholesterol biosynthesis, protein ubiquitination and oxidation of fatty acid. The top upstream regulators in this contrast were *HNF4A, E2F1, PPARA, NFE2L2* and *E2F4*. “Cell Death and Survival” (434 AR-DEGs) and “Cell Cycle” (270 AR-DEGs) were the most populated Molecular and Cellular Functions identified by IPA. Under the “Physiological System Development and Function” category, 278 AR-DEGs were assigned by IPA to the “Organismal Survival” subcategory. The top up-regulated genes in this contrast included *IGFBP2, ADSL, LDHB* and mannose binding lectin 2 (*MBL2*), whereas *THRSPA, ME1, SCD* and galectin 2 (*LGALS2*) were the highest expressed AR-DEGs in the D3REFED24h treatment.

**Table 4:** IPA summary of liver transcriptomes in hatcling chicks–D2FAST48h vs. D3REFED24h contrast

Top Canonical Pathways	*p*-value	Overlap	Ratio
Tryptophan Degradation III	4.58E-12	56.0%	14/25
NRF2-mediated Oxidative Stress Response	4.30E-09	17.1%	33/193
Super-pathway of Cholesterol Biosynthesis	8.44E-09	42.9%	12/28
Protein Ubiquitination Pathway	1.42E-08	14.7%	39/265
Fatty Acid -oxidation I	5.11E-08	37.5%	12/32
Top Upstream Regulators	***p*-value of overlap**	**# Target genes**	
HNF4A	5.44E-22	224	
E2F1	6.07E-19	81	
PPARA	9.50E-19	77	
NFE2L2	4.82E-16	68	
E2F4	7.81E-16	48	
Top Molecular and Cellular Functions	***p*-value**	**# Genes**	
Cell Death and Survival	3.73E-04 - 9.50E-20	434	
Cell Cycle	3.58E-04 - 7.37E-18	270	
Cellular Assembly and Organization	2.92E-04 - 6.25E-17	103	
DNA Replication, Recombination, and Repair	3.58E-04 - 6.25E-17	176	
Amino Acid Metabolism	2.69E-04 - 2.67E-12	38	
Physiological System Development and Function	***p*-value**	**# Genes**	
Organismal Survival	6.21E-09 - 2.57E-09	278	
Digestive System Development and Function	1.35E-04 - 1.47E-07	82	
Hepatic System Development and Function	4.62E-05 - 1.47E-07	53	
Organ Morphology	3.43E-05 - 1.47E-07	52	
Organismal Development	4.62E-05 - 1.47E-07	65	
Up-regulated genes	**FAST48h-REFED24h**	**Down-regulated genes**	**FAST48h-REFED24h**
*IGFBP2*	3.19	*THRSPA*	−5.99
*ADSL*	3.19	*ME1*	−4.84
*LDHB*	2.90	*SCD*	−4.72
*MBL2*	2.85	*LGALS2*	−3.65
*ALB*	2.71	*CYP51A1*	−3.30
*CYP4A22*	2.66	*PLIN2*	−3.26
*UPP2*	2.61	*FADS2*	−3.24
*BHMT*	2.56	*CDK1*	−3.06
*CYP3A7*	2.40	*RRM2*	−3.03
*NEFH*	2.39	*CDCA3*	−2.93

Ingenuity Pathway Analysis (IPA) was used for functional analysis of 1198 “Analysis Ready” (AR)-DEGs from the D2FAST48h vs. D3REFED24h contrast. The top 10 up-regulated and down-regulated AR-DEGs are presented along with their respective log2 ratio of treatment conditions

**Fig. 7:**
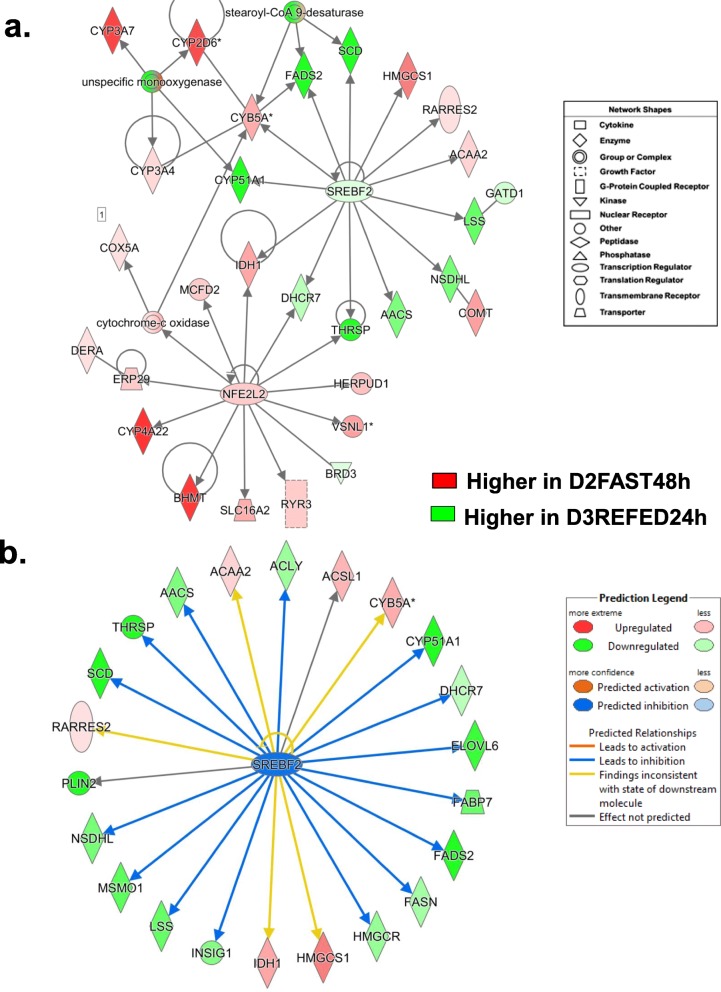
This “Lipid Metabolism” network (Panel **a**) is centered on interactions of three transcription factors, thyroid hormone-responsive Spot14 protein (*THRSP*), sterol response element binding factor 2 (*SREBF2*) and nuclear factor, erythroid 2-like 2 (*NFE2L2*) and their direct target genes from the D2FAST48h vs. D2REFED4h contrast. IPA predicted *activation* (orange lines and arrows) or *inhibition* (blue lines) of direct targets of *SREBF2* identified in the D2FAST48h vs. D2REFED4h contrast (Panel **b**). Of the 22 direct target genes identified, only six AR-DEGs were expressed at higher levels in liver of D2FAST48h chicks. As such, Ingenuity predicts that *SREBF2* should be inhibited (blue symbol), which would lead to inhibition (blue arrows/edges) of 16 DEGs (green symbols) that control lipogenesis under the direction of the most highly-expressed gene in liver of fed or refed cockerels— the lipogenic transcription factor *THRSPA*

The gene network presented in Fig. 7a was functionally annotated by IPA as “Lipid Metabolism” and centered on interactions of *SREBF2* and *NFE2L2* with their direct target AR-DEGs, including three shared genes [*THRSP(A),* 7-dehydrocholesterol reductase (*DHCR7*) and isocitrate dehydrogenase (NADP(+)) 1, cytosolic (*IDH1*)]. Most direct targets of *SREBF2* are lipogenic and expressed at higher levels in liver of the D3REFED24h chicks, including *CYP51A, FASD2, SCD*, lanosterol synthase (*LSS*), NAD(P) dependent steroid dehydrogenase-like (*NSDHL*), acetoacetyl-CoA synthetase (*AACS*), *THRSP,* and *DHCR7.* However*,* five AR-DEGs targets of SREBF2 are up-regulated genes (red symbols) in this network, namely *IDH1*, cytochrome b5 type A (*CYB5A*), *HMGCS1*, retinoic acid receptor responder 2 (*RARRES2*; or chemerin, an adipokine) and acetyl-CoA acyltransferase 2 (*ACAA2*), the enzyme that catalyzes the final reaction in the fatty acid β-oxidation pathway. In contrast, most direct targets of *NFE2L2* were up-regulated by the D2FAST48h treatment, especially two very abundant target AR-DEGs [*CYP4A22* and betaine--homocysteine S-methyltransferase (*BHMT*)]. Ingenuity Upstream Regulator Analysis identified 22 direct targets of *SREBF2* from this contrast (Fig. 7b), 6 AR-DEGs were up-regulated, while 22 genes were down-regulated or expressed higher in the D3REFED24h treatment group. Ingenuity predicts that *SREBF2* activity should be inhibited, which would lead to inhibition of 15 direct target genes as indicated by the blue arrows.

**Fig. 8:**
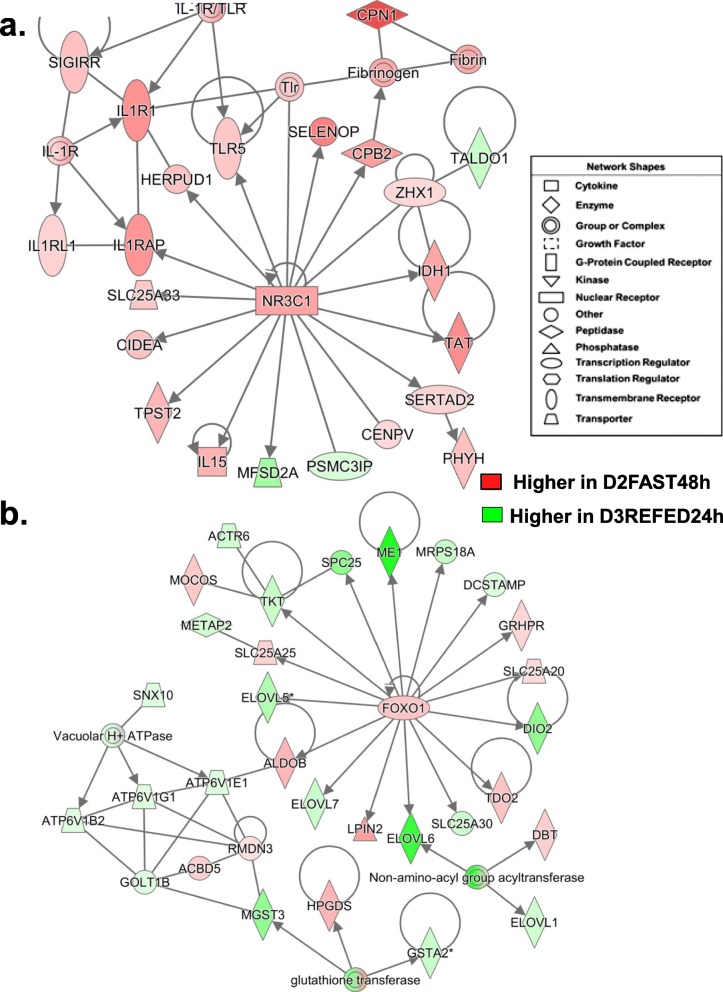
A gene interaction network was identified in the D2FAST48h vs. D3RERFED24h contrast and functionally annotated by IPA as related to “Nutritional Disease” (Panel **a**). This gene network was centered on the interaction of the glucocorticoid receptor (*NR3C1*) and 16 direct target genes, including several components of the innate immune response/ inflammation (*IL15, IL1RAP, TLR5* and *HERPUD1*) and regulators of lipolysis (*SERTAD2* and *CIDEA*); all of which were highly expressed in liver of D2FAST48h chicks. Panel **b** shows another gene network related to “Lipid Metabolism” that involves the transcription factor forkhead 1 (*FOX01)* and its 16 direct targets that interact, via *ALDOB,* with three ATPases and glutathione transferases (*GSTA2*, *MGST3* and *HPGDS*). The majority of the genes in this network are expressed higher in liver of D3REFED24h chicks, except *FOX01, GRHPR, SLC25A20, IDO2, ALDOB* and *SLC25A25*, which are up-regulated by the D2FAST48h treatment

Another gene network identified by IPA in the D2FAST48h vs. D3REFED24h contrast focused on the *GCR* (*NR3C1*) and was functionally annotated as “Nutritional Disease” (Fig. 8a). The *NR3C1* and all of its direct target genes, except *MFSD2A* and PSMC3 interacting protein (*PSMC3IP*), were expressed at higher levels in liver of D2FAST48h chicks. Three direct targets of *NR3C1* [interleukin 1 receptor accessory protein (IL1RAP), homocysteine inducible ER protein with ubiquitin like domain 1 (*HERPUD1*) and *TLR5*] interact with components of the ILR1 complex [interleukin 1 receptor type 1 (*IL1R1*), interleukin 1 receptor like 1 (*IL1RL1*), single Ig and TIR domain containing (*SIGIRR*). Another direct target gene, carboxypeptidase B2 (*CPB2*), encodes the carboxypeptidase that inhibits fibrinolysis, while promoting fibrinogenesis and the carboxypeptidase N subunit 1 (*CPN1*) gene. Additional up-regulated direct targets of *NR3C1* include, selenoprotein P (*SELENOP*), zinc fingers and homeoboxes 1 (*ZHX1*), *IDH1, TAT, SERTAD2,* centromere protein V (*CENPV*), *IL15*, tyrosylprotein sulfotransferase 2 (*TPST2*), cell death-inducing DFFA-like effector a (*CIDEA;* an activator of apoptosis), and solute carrier family 25 member 33 (*SLC25A33*), which encodes the mitochondria pyrimidine nucleotide transporter. Interestingly, two targets of NR3C1 (*SERTAD2* and *CIDEA*) in this network are important regulators of lipolysis and thermogenesis, whereas the direct target of the TF *SERTAD2*, phytanoyl-CoA 2-hydroxylase (*PHYH*), catalyzes the alpha-oxidation of 3-methyl-branched fatty acids.

The second gene network (Fig. 8b) was annotated by IPA as “Lipid Metabolism” and was centered on interactions of the TF forkhead box O1 (*FOXO1*) with its 16 direct target genes. One up-regulted target gene, aldolase, fructose-bisphosphate B (*ALDOB*), was linked to components of vacuolar ATPase [ATPase H+ transporting V1, subunit B2 (ATP6V1B2), subunit G1 (ATP6V1G1) and subunit E1 (ATP6V1E1)] and glutathione transferase [microsomal glutathione S-transferase 3 (*MGST3*), glutathione S-transferase alpha 2 (*GSTA2*) and hematopoietic prostaglandin D synthase (*HPGDS*)]. Six direct targets of *FOXO1* were up-regulated in the D2FAST48h vs. D3REFED24h contrast [glyoxylate and hydroxypyruvate reductase (*GRHPR*), indoleamine 2,3-dioxygenase 2 (*IDO2*), *LPIN2*, *ALDOB,* and *SLC25A25*], while 10 AR-DEGs were expressed higher in liver of D3REFED24h cockerels [*ME1*, mitochondrial ribosomal protein S18A (*MRPS18A*), dendrocyte expressed seven transmembrane protein (*DCSTAMP*), *DIO2*, solute carrier family 25 member 30 (*SLC25A30*), ELOVL fatty acid elongase 6 (*ELOVL6*), *ELOVL*7, *ELOVL*5, transketolase (*TKT*), and NDC80 kinetochore complex component (*SPC25*). Five additional genes were indirectly related to *FOXO1* [dihydrolipoamide branched chain transacylase E2 (*DBT*), *ELOVL1*, methionyl aminopeptidase 2 (*METAP2*), molybdenum cofactor sulfurase (*MOCOS*), and ARP6 actin related protein 6 homolog (*ACTR6*)].

Additional file 5 provides annotated lists of AR-DEGs assigned by IPA to canonical pathways and biological processes over-represented by the D2FAST48h vs. D3REFED24h contrast (see Table 4). An almost equal number of transcription factors were up-regulated (9 AR-DEGs) or down-regulated (8 AR-DEGs) in this contrast. The major canonical pathways over-represented in this contrast were “Protein Ubiquitination” (1 up-regulated/36 downregulated), Nuclear Regulatory Factor 2 “(NRF2)-Mediated Oxidative Stress Response” (16 up-regulated/17 downregulated), “Fatty Acid Metabolism” (26 up-regulated/3 downregulated), “Acute Phase Response” (20 up-regulated/4 downregulated), “PPARA/RXRA Activation” (13 up−/6 down-regulated), “LXR/RXR Activation” (15 up−/3 down-regulated), “Fatty Acid β-Activation” (11 up−/1 down-regulated), and “Coagulation System” (8 up-regulated only).

#### Effects of re-feeding for 48 h [D2FAST48h vs. D4REFED48h contrast]

The IPA summary of liver transcriptomes in the D2FAST48h vs. D4REFED48h contrast is presented in Table 5. The top canonical pathways identified in this contrast were related to cholesterol biosynthesis, tryptophan degradation, sirtuin signaling, and inhibition of retinoid X receptor (RXR) function. The top five upstream regulators in this contrast were PPARA, NFE2L2, HNF4A, SREBF2 and PPARG coactivator 1 alpha (*PPARGC1A*), a major regulator of energy metabolism. The major “Molecular and Cellular Functions” were “Lipid Metabolism” (181 AR-DEGs), “Molecular Transport” (190 AR-DEGs), and “Small Molecule Biochemistry” (237 AR-DEGs). The top “Physiological System Development and Function” subcategories of IPA represented by AR-DEGs from this contrast were related to the digestive and hepatic systems, embryonic and organismal development, and organ morphology. The top five up-regulated genes in this contrast were *IGFBP2, ADSL, CYP3A7, CYP4A22* and *BHMT*; whereas, the top five down-regulated genes were *THRSPA, ME1, SCD, LGALS1 and PLIN2*, these lipogenic genes were expressed higher in liver of the D4REFED48h chicks.

**Table 5:** IPA summary of liver transcriptomes in hatchling chicks-D2FAST48h vs. D4REFED48h contrast^a^

Top Canonical Pathways	*p*-value	Overlap	Ratio
Super-pathway of Cholesterol Biosynthesis	7.68E-10	39.3%	11/28
Tryptophan Degradation III	3.74E-09	40.0%	10/25
Sirtuin Signaling Pathway	6.93E-08	10.3%	30/292
Cholesterol Biosynthesis I-II	6.99E-08	53.8%	7/13
LPS/IL-1 Mediated Inhibition of RXR Function	5.58E-07	10.8%	24/222
Top Upstream Regulators predicted by IPA	***p*-value of overlap**	**# Target genes**	
PPARA	1.41E-20	62	
NFE2L2	1.09E-14	50	
HNF4A	3.61E-12	134	
PPARGC1A	4.21E-12	34	
SREBF2	5.77E-12	19	
Top Molecular and Cellular Functions	***p*-value**	**# Genes**	
Lipid Metabolism	1.23E-03 - 6.04E-16	181	
Molecular Transport	1.28E-03 - 6.04E-16	190	
Small Molecule Biochemistry	1.28E-03 - 6.04E-16	237	
Vitamin and Mineral Metabolism	1.14E-03 - 5.30E-14	64	
Amino Acid Metabolism	1.14E-03 - 3.22E-10	38	
Physiological System Development and Function	***p*-value**	**# Genes**	
Digestive System Development and Function	1.14E-03 - 6.09E-09	87	
Hepatic System Development and Function	1.14E-03 - 6.09E-09	67	
Organ Morphology	7.07E-04 - 6.09E-09	46	
Organismal Development	1.22E-03 - 6.09E-09	147	
Embryonic Development	1.22E-03 - 3.24E-08	55	
Up-regulated genes	**FAST48h/REFED48h**	**Down-regulated genes**	**FAST48h/REFED48h**
***IGFBP2***	**3.31**	***THRSPA***	**−6.47**
***ADSL***	**3.21**	***ME1***	**−4.97**
***CYP3A7***	**2.51**	***SCD***	**−4.55**
***CYP4A22***	**2.39**	***LGALS2***	**−4.14**
***BHMT***	**2.34**	***PLIN2***	**−3.64**
***AIF1L***	**2.31**	***FADS2***	**−3.86**
***LDHB***	**2.16**	***CYP51A1***	**−3.56**
***ABCG5***	**2.06**	***DIO2***	**−2.71**
***NDRG1***	**2.04**	***ENOVL6***	**−2.59**
***AKR1D1***	**1.97**	***MSMO1***	**−2.58**

^a^Ingenuity Pathway Analysis (IPA) was used for functional analysis of 750 “Analysis Ready” (AR)-DEGs from the D2FAST48h vs. D4REFED48h contrast. The top 10 up-regulated and down-regulated AR-DEGs are presented along with their respective log2 ratio of treatment conditions, where positive numbers indicate higher expression in the D2FAST48h treatment and negative values indicate higher expression in the D4REFED48h treatment condition

**Fig. 9:**
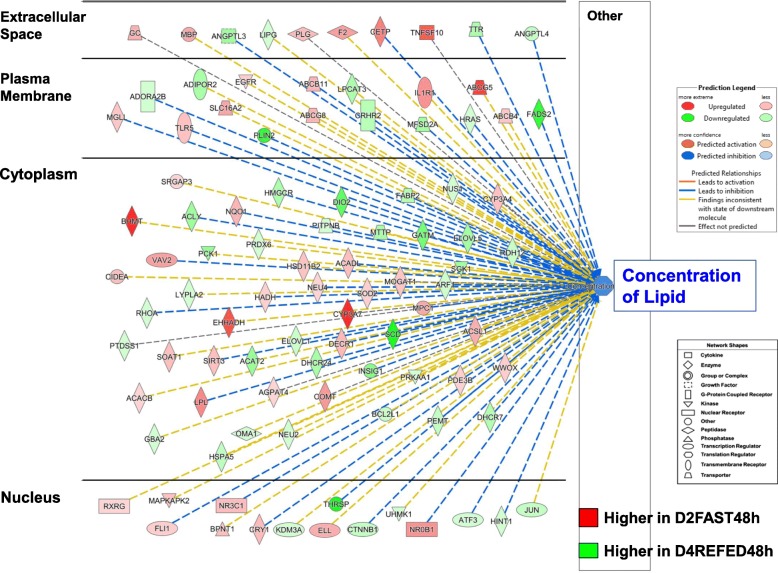
Subcellular distribution of 97 AR-DEGs functionally annotated by IPA as “Concentration of Lipids” from the D2FED48h vs. D4REFED48h contrast. This figure provides an overview of the transcriptional hierarchy of hepatic genes that control the concentration of lipids, which was revealed by the D2FAST48h vs. D4REFED48h contrast. Genes with red symbols are expressed higher in liver of D2FAST48h cockerels, while green symbols indicate higher hepatic expression in the D4REFED48h treatment. A relatively small group of 15 upstream regulators control transcription of numerous metabolic enzymes, transporters, kinases and phosphatases in the cytoplasm, several transmembrane receptors, G-protein-coupled receptors, peptidases and enzymes in the plasma membrane, and even fewer numbers of growth factors, transporters and enzymes in extracellular space. IPA predicts that the concentration of lipid in liver would be inhibited by the eight up-regulated transcription factors as indicated by the dashed blue lines, while yellow dashed lines represent inconsistence between the expected state and observed of the downstream genes. The annotated list of these 97 AR-DEGs controlling concentration of lipid is provided in Additional file 6

The subcellular distribution of 97 AR-DEGs controlling the hepatic concentration of lipid from the D2FAST48h vs. D4REFED48h contrast is presented in Fig. 9. Fifteen nuclear TFs (8 up-regulated in the D2FAST48h group and 7 expressed higher in D4REFED48h chicks) control hepatic lipid concentration via their own interactions and with interactions with 82 AR-DEGs that are found across the cytoplasm, cell membrane and extracellular space (Additional file 6). Genes with red symbols are expressed higher in liver of D2FAST48h chicks, whereas green-colored gene symbols indicate higher expression in liver of D4REFED48h chicks. Ingenuity predicts inhibition of the concentration of lipid in the D2FAST48h vs. D4REFED48h contrast, as expected from the D2FAST48h/D4REFED48h expression ratios. The dashed blue lines indicate AR-DEGs that are predicted by IPA to inhibit lipid concentration, while yellow dashed lines indicate an inconsistent finding according to IPA’s expected expression of downstream genes. Up-regulated cytoplasmic genes (red symbols), expressed at higher levels in liver of D2FAST48h chicks, are associated with lipolysis *(LPL, CIDEA*), beta-oxidation (*ACADL*, *EHHADH*), glycolysis *(SIRT5)* and synthesis of cholesterol (CYP3A7) and phospholipids (*AGPAT4, MOGAT1*). On the other hand, several cytoplasmic genes expressed higher in the D4REFED48h treatment, are involved in lipid metabolism (*SCD, INSIG1, ACAT2, HMGCR, FABP2, ELOVL5*). Type 2 deiodinase (*DIO2*) controls generation of metabolically-active T3, which drives activity of *THRSPA*, the major *lipogenic* transcription factor in the chicken. Additional genes found in the plasma membrane (*ADIPOR2, PLIN2, LPCAT3, FADS2*) or extracellular space (*LIPG, ANGPTL3, ANGPTL4*) that support lipid metabolism are also expressed at higher levels in liver of D4REFED48h chicks.

**Fig. 10:**
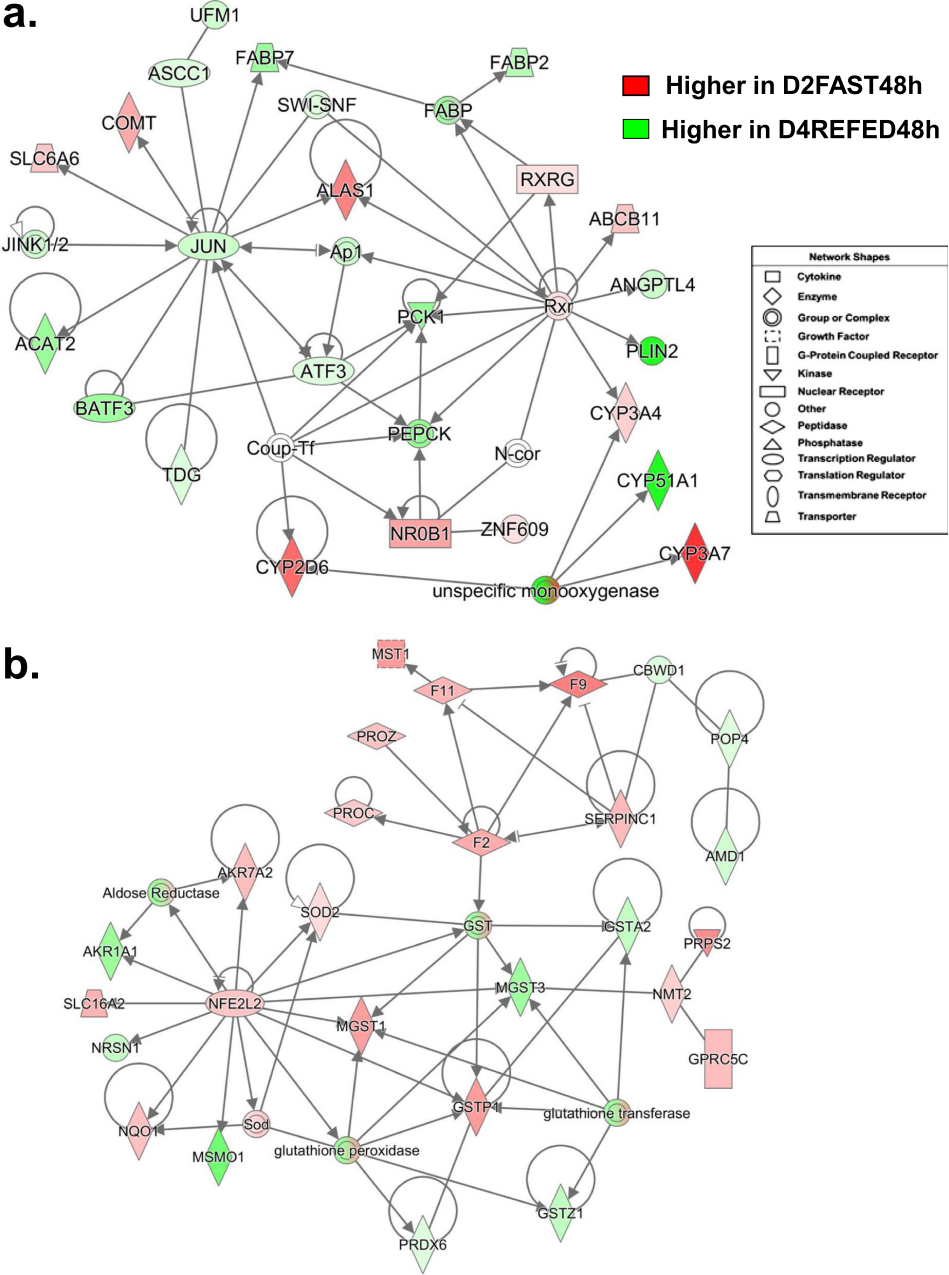
A gene network depicting interactions among six transcription factors (*JUN, RXR, NR0B1, AFT3, BATF3* and *ASCC1*) and their target genes identified from the D2FAST48h vs. D4REFED48h contrast (Panel **a**). Nine genes in this network, annotated by IPA as “Lipid Metabolism/Molecular Transport”, were expressed higher in liver of chicks under the D2FAST48h treatment, while 15 genes were expressed at higher levels in the D4REFED48h chicks. Ingenuity Up-stream Regulator Analysis (Panel **b**) predicts that Jun proto-oncogene, AP-1 transcription factor subunit (*JUN*) would be inhibited (blue gene symbol) and that seven of the 33 direct targets of JUN would be inhibited (blue arrows and green gene symbols), whereas only two target genes [5′-aminolevulinate synthase 1 (*ALAS1*) and myelin basic protein (*MBP*)] were predicted to be actively inhibited by JUN (blunt orange line). Another ligand-activated transcription factor, thyroid hormone-receptor beta (*THRB*), forms heterodimers with retinoid X receptor gamma (*RXRG*). Although not an AR-DEG itself, Ingenuity predicts that THRB would be inhibited which would lead to inhibition of six direct target genes (*CTNNB1, ME1, PCK1, PPPCA, THRSPA* and *YWHAE*), while two target genes (*EGFR* and *EHHADH*) would be actively blocked (blunt orange edge)

A gene interaction network functionally annotated by IPA as “Lipid Metabolism” and “Molecular Transport” was centered on the interactions of four upstream regulators identified in the D2FAST48h vs. D4REFED48h contrast (Fig. 10a). Four transcription factors [Jun proto-oncogene, AP-1 transcription factor subunit (*JUN*), basic leucine zipper ATF-like transcription factor 3 (*BATF3*), activating signal co-integrator 1 complex subunit 1 (*ASCC1*), and activating transcription factor 3 (*ATF3*)] were expressed higher in liver of D4REFED48h chicks. In contrast, two transcription factors were up-regulated in this contrast [retinoid X receptor gamma (*RXRG*) and nuclear receptor subfamily 0 group B member 1 (*NR0B1*; a dominant-negative regulator of transcription)]. The transcription factor *JUN* directly interacts with six down-regulated AR-DEGs [*ASCC1*, fatty acid binding protein 7 (*FABP7*), TDG, BATF3, acetyl-CoA acetyltransferase 2 (*ACAT2*)] and three down-regulated AR-DEGs [5′-aminolevulinate synthase 1 (*ALAS1*), solute carrier family 6 member 6 (*SLC6A6*) and catechol-O-methyltransferase (*COMT*)]. *RXRG* and the *RXR* complex were up-regulated and interact with the downregulated *FABP* complex, which includes *FABP2* and *FABP7*, phosphoenolpyruvate carboxykinase 1 (*PCK1* or *PEPCK*), *ANGPTL4* and *PLIN2,* also down-regulated. Two direct target genes of the *RXR* complex [ATP binding cassette subfamily B member 11 (*ABCB11*) and *CYP3A4*] were up-regulated, while two additional up-regulated monoxygenases (*CYP2Q6* and *CYP3A7*) and the down-regulated *CYP51A1* were indirect members of this network.

The gene network shown in Fig. 10b was functionally annotated as “Hematological System” and composed of three components: the transcription factor *NFE2L2* and its target genes, five up-regulated coagulation factors [macrophage stimulating 1 (*MST1*), coagulation factor II, thrombin (*F2*), coagulation factor IX (*F9*), coagulation factor XI (*F11*), protein C, inactivator of coagulation factors Va and VIIIa (*PROC*), protein Z, vitamin K dependent plasma glycoprotein (*PROZ*) and serpin family C member 1 (*SERPINC1*; or anti-thrombin 3, AT3)] and six glutathione enzymes. Five direct targets of *NFE2L2* were up-regulated in this contrast [aldo-keto reductase family 7 member A2 (*AKR7A2*), superoxide dismutase (*SOD2*), microsomal glutathione S-transferase 1 (*MGST1*), NAD(P)H quinone dehydrogenase 1 (*NQO1*), solute carrier family 16 member 2 (*SLC16A2*; a thyroid hormone carrier protein)], while three target AR-DEGs were down-regulated [MSMO1, neurensin 1 (*NRSN1*) and aldo-keto reductase family 1 member A1 (*ARK1A1*)]. Other members of the glutathione enzyme complex include peroxiredoxin 6 (*PRDX6*), glutathione S-transferase zeta 1 (*GSTZ1*), glutathione S-transferase pi 1 (*GSTP1*), microsomal glutathione S-transferase 3 (*MGST3*), glutathione S-transferase alpha 2 (*GSTA2*), N-myristoyltransferase 2 (*NMT2*), phosphoribosyl pyrophosphate synthetase 2 (*PRPS2*) and G protein-coupled receptor class C group 5 member C (*GPRC5C*; or retinoic acid-inducible gene 3, *RAIG3*).

### Quantitative reverse transcription PCR (qRT-PCR) analysis: verification of differential gene expression of 16 candidate genes

**Fig. 11:**
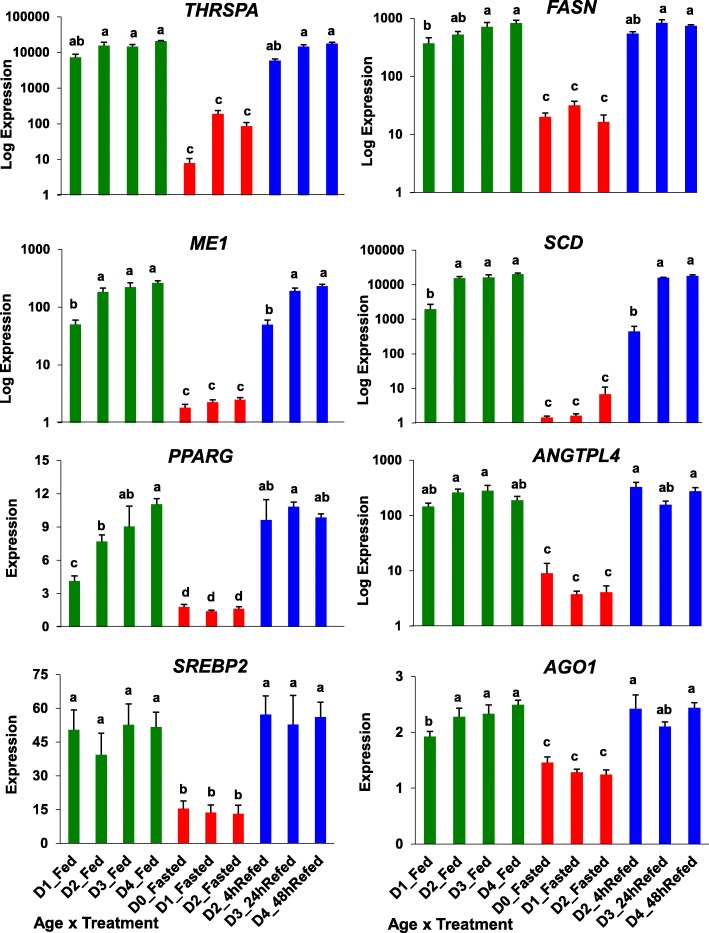
Verification of differential expression of eight *lipogenic* genes using qRT-PCR analysis. Nutritional state is indicated by bar color, where green = fed state, red = fasted, and blue = refed conditions. Values represent least-square means (LSM) and their standard error (LSE) of normalized expression levels of five cockerels (biological replicates) and two technical replicates. Expression levels, determined by qRT-PCR analysis, were normalized using the geNorm procedure in qBase software [59]. Values possessing different superscripts are significantly different as determined by analysis of variance (ANOVA) using the general linear models (GLM) procedure in Statistical Analysis System (SAS) software and with mean separation using Tukey’s Studentized Range Test

The independent measurement of gene expression of eight lipogenic genes by qRT-PCR analysis is presented in Fig. 11. The expression of these metabolic genes was greatly depressed by fasting for 4, 24 or 48 h and restored after refeeding (4, 24 or 48 h), whereas only *PPARG* exhibited a progressive increase in hepatic expression with age in the fed state. Although not a metabolic gene per se, argonaute 1 (*AGO1*), which is involved in RNA silencing, had the same expression pattern as the three transcription factors and four metabolic enzymes presented in this figure. The ANOVA showed a significant (*P* ≤ 0.001) treatment effect (fed, fasted or refed) for each of these 8 “candidate” genes. Five of these lipogenic genes showed a significant (*P* ≤ 0.05) treatment x age interaction (*THRSPA, ME1, SCD, PPARG* and *ANGTPL4*). Furthermore, a log10-scale difference in expression was found for six genes (*THRSPA, FASN, ME1, SCD, PPARG* and *ANGTPL4*).

**Fig. 12:**
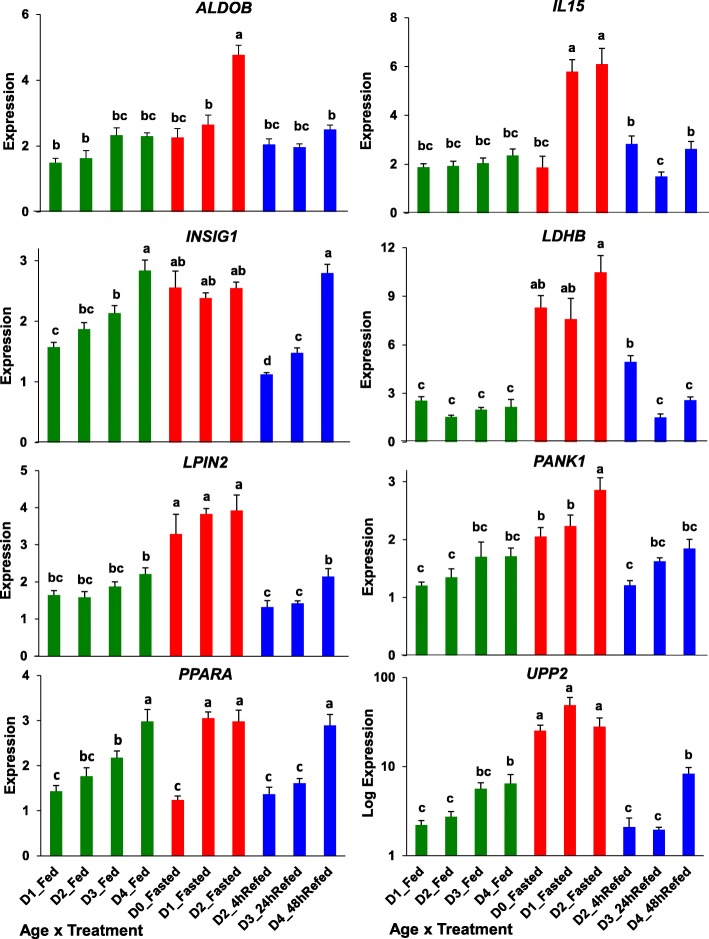
Verification of differential expression of eight *lipolytic* genes using qRT-PCR analysis. Nutritional state is indicated by bar color, where green = fed, red = fasted and blue = refed condition. Values represent least-square means (LSM) and their standard error (LSE) of normalized expression of five cockerels (biological replicates) analyzed in duplicate. Expression levels, determined by qRT-PCR analysis, were normalized using the geNorm procedure in qBase software [59]. Values possessing different superscripts are significantly different as determined by analysis of variance (ANOVA) using the general linear models (GLM) procedure in Statistical Analysis System (SAS) software and with mean separation using Tukey’s Studentized Range Test

The next figure (Fig. 12) presents the qRT-PCR analysis of 8 “lipolytic” genes whose expression responds to either fasting (*ALDOB, IL15, LDHB, LIPIN2, PANK1, PPARA* and *UPP2*) or refeeding after a 48 h fast (*INSIG2*). The only gene to present a log10-scale difference in expression was *UPP2*. ANOVA showed that all eight genes had significant main effects of treatment (FED, FAST, REFED) and age (D0-D4), while only four genes (*ALDOB, IL15, INSIG1* and *LDHB*) showed a significant treatment X age interaction. Three genes demonstrated a clear progressive increase in expression with age in the feed state (*INSIG2, PPARA* and *UPP2*).

Our qRT-PCR analysis also included four additional genes that were not shown in Fig. 11 and Fig. 12 (Additional file 7). FAT atypical cadherin 1 (*FAT1*) and glyceraldehyde-3-phosphate dehydrogenase (*GAPDH*) were determined as DEGs by both microarray and qRT-PCR analyses (Panel a). *FAT1* expression was higher in the fed state, depressed by fasting and restored with refeeding. FAT1 is a tumor suppressor and involved in WNT signaling. Both *FAT1* and *GAPDH* showed a progressive increase with age in the fed state, both were depressed in the fasted state and recovered on D4 after being refed for 48 h. *GAPDH* was originally included in qRT-PCR analysis as potential invariant gene; however, *GAPDH* was rejected for normalization by the geNorm software. Two invariant genes (Panel b), cytochrome c oxidase subunit 7A2 like (*COX7A2L*) and ribosomal protein L14 (*RPL14*), were used by geNorm software for normalization of qRT-PCR expression levels of candidate genes.

A Pearson’s Correlation Analysis was made of the transcriptional responses of 11 candidate genes as determined by microarray and qRT-PCR analyses (Additional file 8). Since the statistical analysis of microarray data was based on normalized differential log2 expression ratios from pair-wise contrast of fasting and refeeding conditions, normalized expression levels established by qRT-PCR analysis were used to create appropriate pairwise log2 ratios for comparison across seven treatment conditions. The only candidate that failed to show a positive correlation between microarray and qRT-PCR analysis was insulin induced gene 1 (*INSIG1*), which was not included in the Pearson’s Correlation Analysis. The Pearson’s Correlation Analysis was completed for 11 candidate genes and the resultant Pearson’s Correlation coefficient was used to verify differential gene expression from two analytical methods. The average Pearson coefficient across the 11 candidate genes was r = 0.970, which indicates a highly significant (*P* ≤ 0.001) correlation between gene expression levels obtained from microarray and qRT-PCR analyses. Unfortunately, two important lipid metabolism genes, *FASN* (RIGG07906 oligo) and *PPARA* (RIGG05780 oligo), appeared quite variable in the present microarray study and, therefore, were not identified as DEGs by statistical analysis, although qRT-PCR analysis did reveal them as DEGs.

## Discussion

The present transcriptional analysis of liver in newly-hatched chicks during a fasting-refeeding perturbation provides new insight into transcriptional control over robust *homeorhetic* adjustments made during the switch from lipolysis to lipogenesis. When provided with high-carbohydrate protein-enriched feed, the hatchling chick is able to double its body weight in a mere 4 days (see Fig. 1). Although newly-hatched chicks can survive for several days without feed by utilizing lipids from the absorbed yolk sac, prolonged fasting for the first 48 h post-hatching prevents recovery of body weight gain, even after 2 days after refeeding (D4). Pathway and functional analyses of hepatic DEGs have identified several major transcription factors that are interconnected and interdependent. These transcription factors appear to provide dynamic *homeorhetic* control over energy metabolism during the switch in metabolism from lipolysis to lipogenesis, as we have shown here during the switch from fasted to refed states in newly-hatch chicks.

### Transcriptional regulation of metabolism during the fasting-refeeding perturbation

Our initial visual examination of hepatic gene expression using hierarchical and K-means clustering clearly shows two distinct patterns, where expression of a large group of lipogenic genes was depressed by fasting and quickly restored after refeeding, or remained high in the fully-fed state. Another group of largely lipolytic genes showed the opposite pattern with highest abundance during fasting and lowest expression after refeeding. This opposing pattern of gene expression suggests a mechanism of reciprocal inhibition of lipolytic and lipogenic genes by the predominant transcription factors controlling either metabolic state, as discussed below.

Functional and gene network analyses of AR-DEGs, derived from multiple pair-wise contrasts of fasting-refeeding conditions, revealed several major transcription factors in liver that respond strongly to the metabolic perturbation of fasting and re-feeding. Our study shows that *THRSPA* appears to be a principal regulator of multiple lipogenic genes (*SCD, ME1, FASN, FADS2, PLIN2, ELOVL6, MSMO1, NSDHL* and *INSIG1*) expressed in adipose tissue [13–15]. And, as we have recently shown in liver of late embryos and hatchling chicks during the natural perturbation of hatching, *THRSPA* is also the key transcriptional regulator of the switch in metabolism from ectotherm*y* to endothermy [5]. This genome-wide study of liver transcriptomes in newly hatched chicks has also identified *THRSPA* as the most abundant lipogenic transcription factor expressed. Furthermore, we have identified several additional DEGs (i.e., *SREBF2, PPARG, PPARA, JUN, FOX01, CTNNB1, NR3C1, SERTAD2, SIRT3* and *SIRT5*) that contribute to the metabolic switch from fasting (lipolysis) to refed (lipogenesis) states. These transcription factors can be functionally divided into two groups *lipolytic* (*PPARA, NR3C1, SERTAD2, SIRT3, SIRT5, RXR, NROB1* and *FOX01*) and *lipogenic* (*THRSPA*, *SREBF2, PPARG, CTNNB1* and *JUN*). Apparently, a handful (5–15) of key transcription factors regulates a larger number (100) of downstream genes involved in the synthesis or concentration of lipid in liver of newly-hatched chicks.

In mammals, the *THRSP* gene is highly expressed in lipogenic tissue (liver, fat, mammary gland, ovary) and regulated by several hormones and metabolic factors with common response elements in its promotor region (ChoRE, SRE, LXRE and PPRE), hormone-activated nuclear receptors (*THRB, RXR, LXR, VDR, ESR,* and *GCR*), and other transcription factors (*SREBF2, PPARG, PPARA, HNF4A, CREBP2, and ChREBP*). Interestingly, insulin deprivation in fed chickens depresses hepatic expression of *THRSPA, ERG1, PPARG, DIO2, GK* and *FASN* [16]. Furthermore, the insulin-induced gene 1 (*INSIG1*) was highly expressed in liver of our fed and refed hatchling chicks. In mammals, *INSIG1* is a known regulator of lipid metabolism [17]. Presently, *THRSPA* and *DIO2* were the highest expressed hepatic genes in the D1FED24h vs. D1FAST24h contrast (see Table 3). Therefore, generation of metabolically-active T_3_ from enhanced DIO2 activity and co-expression of *THRB* could support enhanced *THRSPA* expression, since its promotor contains three synergistic thyroid hormone response elements (TRE) [18], activated by the ligand-bound heterodimer THRB-RXR. Furthermore, paralogs of THRSP [*THRSPΑ*, *THRSPΒ*; Spot 14 (*S14*); *THRSP-like,* (*THRSPL*) or *MIG12 or S14R*)] share common response elements (RE) in their promotor regions and have overlapping roles in regulating lipogenesis [19] and lipid metabolism. For example, the heterodimer S14-S14R is considered a strong ‘metabolic inhibitor’ of human acetyl-CoA carboxylase 2 (ACACA) [20], which catalyzes the rate-limiting step in biosynthesis of fatty acids. Specifically, the S14-S14R heterodimer exerts a double action by preventing dimerization of ACACA isoforms and by blocking catalytic activity of the ACACA enzyme [20]. Thus, some actions of *THRSPA* on lipogenesis in chickens are likely mediated by dimers of THRSP paralogs. However, the exact mechanism(s) by which *THRSPA* affects the expression or activity of other transcription factors or downstream metabolic genes in the chicken remains unclear. Further investigation will be required for elucidation of the exact mechanisms by which *THRSPA* promotes lipogenesis and for acknowledgement of *THRSPA* as the key *lipogenic* transcription factor regulating lipid metabolism in the chicken.

### Upstream regulators of the metabolic switch from lipolysis (fast) to lipogenesis (refed; fed)

The pair-wise contrasts made across 10 nutritional conditions proved useful in detecting the hierarchy of transcriptional control over responses of hundreds of downstream DEGs involved in the *homeorhetic* shift from lipolytic to lipogenic metabolism. Many ligand-activated nuclear factors interact with THRSPA and other transcription factors in control of expression of their gene targets, which facilitate the *homeorhetic* switch from fasting to refeeding in newly-hatched chicks. During prolonged fasting, increased hepatic sirtuins (*SIRT1, SIRT5)* and *SERTAD2* stimulate lipolysis, glycolysis and gluconeogenesis via enhanced expression of numerous genes, including *CPT1A, NR1H4, PDK4, PPARGC1A* and *ADIPOQ*, as we found during the embryo-to-hatchling transition [5] and presently during a bout of fasting and refeeding in D0-D4 hatchlings. The deacetylase SIRT5 regulates glycolysis and β-oxidation of fatty acids [21], while SERTAD2 also controls lipolysis, oxidative metabolism and thermogenesis [22–24]. We recently discovered *SERTAD2* as a novel co-regulator of lipid catabolism, highly expressed in liver of E16 and E18 embryos, with known interactions with other lipogenic transcription factors (*PPARA, PPARGC1A, SIRT1* and *NR1H4*) [5]. For example, *NR1H4* [or farnesoid X receptor (*FXR*)] is a ligand-activated TF, which regulates hepatic bile acid synthesis, while the co-regulator *PPARGC1A* interacts with other transcription factors (*PPARA, PPARG*, and *THRSPA*). The glucocorticoid receptor (*GCR*; or *NR3C1*) was also highly expressed during prolonged fasting; *NR3C1* interacts negatively and positively with multiple transcription factors (*THRSPA, SREBF2, PPARA, PPARD*, and *SERTAD2*) and their downstream target genes to promote lipolysis and gluconeogenesis, while inhibiting lipogenic and adipogenic pathways. Our present example of *homeorhetic* control over lipid metabolism in newly hatched chickens is supported by the inverse (reciprocal) relationship found between hepatic expression of *LXR* and *THRSPA* in hatched chicks vs. week-old fed chicks [10]. The expression of *LXR* was reduced in D7 fed chicks, whereas the expression of lipogenic transcription factors (*THRSPA, SREBP2* and *ChREBP*) and five lipogenic enzymes (*SCD, ME1, FASN, ACACA* and *ACLY*) was much greater in liver of fed one-week-old chicks. Further, LXR is a binding partner with RXR, while RXR forms heterodimers with other ligand-activated nuclear factors (THRB and PPARG), all of which bind to their respective REs on the THRSP promotor. The chicken xenobiotic receptor (*CXR*) is closely related to PXR and CAR orthologs in mammals [25] and is capable of constitutively activating expression of *THRSP*. The ability of RXR to form homeodimers and heterodimers with several nuclear receptors has prompted the view that RXR acts as a ‘promiscuous’ regulator of nuclear receptors [26, 27].

We found higher expression of corticotropin-releasing hormone receptor 2 (*CRHR2*) in liver of D2FAST48h chicks. Exogenous CRH in hatchling chicks enhances expression of β-oxidation enzymes to support thermogenesis [28]. Other transcription factors that are highly expressed during fasting, include *NR0B1* (a dominant-negative regulator of RXR), *RXR*, *FOXO1* (a promotor of insulin signaling [29]), and *NFE2L2* (a negative regulator of fatty acid synthesis) [30]. In our present study, the expression of *FOXO1* was highest with prolonged fasting and showed direct actions with many lipid metabolism enzymes (see Fig. 8). In mammals, *NFE2L2* is a suppressor of adipocyte lipolysis [31]; our study revealed direct interaction of *NFE2L2* with *SREBF2* and *THRSPA* in regulating lipid metabolism (see Fig. 8), while *NFE2L2* was central to a gene interaction network (Fig. 10b) involving glutathione metabolism and the up-regulation of multiple hemostatic factors (*F2, F9, F11, PROC, PROZ* and *SERPINC1*) in liver of the D2FAST48h chicks when compared to D4REFED48h chicks.

In the present study, the adipokine *RARRES2* was up-regulated by prolonged fasting (see Figs. 5 and 7) and regarded as a direct target of *SREBF2*. We first identified chemerin or *RARRES2* as a novel chicken adipokine that was highly expressed in abdominal fat of lean line (LL) chickens rather than fat line (FL) chickens [13]. In contrast, *RARRES2* serves as a biomarker of obesity and metabolic syndrome in humans [32–34]. Other adipokines (or hepatokines) that were expressed higher in liver of fully-fed or refed chicks included angiopoietin-like protein 3 (*ANGPTL3*) and angiopoietin-like protein 4 (*ANGPTL4*), which inhibit plasma lipoprotein lipase (LPL) and therefore clearance of triglycerides and phospholipids.

After hatching and consuming its first carbohydrate and protein rich meal, a small number of transcription factors appear to activate lipogenic, adipogenic and thermogenic pathways, which were strongly depressed in embryos. And as emphasized in the transcriptional analysis of liver from newly-hatched (D0) and fed D7 chicks, the hepatic lipogenic program is likely activated by both endocrine and nutrient signals once the chick consumes carbohydrate-rich feed and purges residual yolk lipids [10]. Major lipogenic genes were up-regulated in liver of fed chicks (*THRSPA, SREBP2, SCD, ME1, FASN, ACLY*, *ACACA, PLIN2, INSIG1* and *DHCR24*), whereas delayed feeding for 48 h post-hatch dampens initial up-regulation of these key lipogenic genes. Many of the same transcription factors identified presently by prolonged fasting-refeeding of newly hatched chicks (D0-D4) were also differentially expressed in liver of embryos compared to hatchling chicks [5]. This observation suggests that a common reciprocal *homeorhetic* mechanism controls metabolic adjustments during the embryo-to-hatchling transition or an episode of fasting and refeeding in hatchlings; both perturbations activate the switch from lipid catabolism (embryo) to lipogenesis/thermogenesis (hatchling).

Another hepatic gene that was highly expressed in the D4REFED48h chicks was *DHCR24,* an insulin induced gene that catalyzes the final step in cholesterol biosynthesis. A recent RNA-seq analysis of liver and adipose tissue in chickens has provided avian biologists an extensive functional catalog with co-expression profiles of mRNA and long-non-coding RNAs (lncRNA) [35]. Of particular interest, *DHCR24* and *lncRNA*_*DHCR24* are positively correlated and both co-expressed at high levels in liver of 9-wk-old cockerels that had been fasted for 12 h and then refed for 3 h before sampling [35]. The possibility of bidirectional promoters and *lncRNA*_*DHCR24* regulation of *DHCR24* expression and ultimately cholesterol synthesis, adds a new dimension to the complexity of transcriptional control over lipid metabolism in the chicken. Certainly, microRNAs are involved in the robust homeorhetic control of energy metabolism induced by fasting and refeeding in newly-hatched chicks. This was well demonstrated in a recent paper describing microRNAs in liver of E18-E20 embryos and D0-D3 hatchlings that target key lipid metabolic genes, including *ADIPOR2, ELOVL6, FASN, ME1, SCD, MSMO1, FADS1, FADS2, INSIG2* and *HMGCS1* [6, 36]. The reciprocal expression pattern exhibited by several key lipogenic genes and the targeting microRNAs is intriguing and likely involved in fine tuning of metabolic responses to fasting and refeeding perturbations, as we have presently described in D0-D4 hatchling chicks. The same metabolic genes were also DEGs found in our transcription analysis of the embryo-to-hatchling transition [5] and presently in fed, fasted or refed D1-D4 chicks. An earlier transcriptional analysis of liver in broiler chicks at hatch (D0) and at D7, after D2 fasted chicks were refed on D2, clearly shows *THRSPA* and *SREBP2* are the prominent up-regulated transcription factors, which could control higher expression of many lipogenic enzymes (i.e., *ME1, FASN, SCD, ACAC* and *ACYL*) at 1 week of age (D7) [10].

Presently, our gene interaction networks show *THRSPA* as the ultimate transcription factor controlling lipogenesis in the newly-hatched chicken. The *THRSP* promoter is driven by multiple response elements, including three synergetic-acting TREs [18], ChoRE [37, 38], SRE and LXRE [39], and a RXR response element [40], although RA-activated RXR forms heterodimers with ligand-activated THRB and LXRA to promote action of their respective response elements. The liver activated receptors (LXR-α,-β) are regulators of adipocyte gene expression; and they form heterodimers with other nuclear hormone receptors (LXR-RXR, RXR-THRB) to control lipid metabolism as directed by *THRSP*, a ‘LXR-responsive gene’ in addition to being glucose- and insulin-responsive [41] and A proven master regulator of lipogenesis and adipogenesis. A polyunsaturated fatty acids response region (PUFA-RR) in the rat THRSP promotor inhibits THRSP transcription [42]. The PUFA inhibition of *THRSP* (*S14*) is mediated by SREBP1a binding to the PUFA-RR within the promoter of the rat *S14* gene [43]. In the rat, PPARA also blocks *THRSP* expression by interfering with THRB-T_3_ binding to the multiple TREs in the *THRSP* promoter, rather than via a PPARA response element (PPAR-RE) [44]. These nutrient- and hormone-activated elements are also found in promotors of five major lipogenic enzymes (*SCD, ME1, FASN, ACACA*, and *ACYL*), see Fig. 3 in [10]. The higher expression of ChREBP is interesting since *THRSPA* transcription parallels the enhanced *SREBP2* and *ChREBP* transcription in liver of D7 chicks; and expression of THRSPA itself is activated by a ChoRE within its promotor. These studies further support our long-held idea that *THRSPA* is the principal transcription factor that regulates lipogenesis during an abrupt switch from *lipolytic* to *lipogenic* metabolism. Thus, multiple response elements activated or inhibited by nuclear factors and metabolites control expression of the lipogenic and adipogenic THRSP paralogs and subsequently lipid synthesis.

In chicken liver, *miR-107* targets *FASN* mRNA while other miRNAs target other lipogenic genes, including *SCD, FADS2, LPIN1, ACACA, ACOX1, MSMO1, ELOVL1, ELOVL2, ELOVL5, INSIG1, HMGCS1* and *HMGCR* [6, 36]. Another miRNA, *miR-128-3p*, inhibits adipogenesis by targeting *PPARA* and *SERTAD2*, which results in blocked differentiation of preadipocytes and enhanced lipolysis, respectively [45]. Furthermore, *MiR-451a* directly targets *THRSP* and downstream lipogenic enzymes*,* which reduces lipogenesis and adipogenesis in mammalian models [46]. From these examples, it seems obvious that *micro-RNAs* and *lncRNAs* contribute to fine tuning of *yin-and-yang* adjustments in lipid metabolism. Our present study of fasting and refeeding in newly-hatched chicks has provided ample evidence of *homeorhetic* responses driven by interconnectivity and inter-dependence exhibited by a few transcriptional regulators (*THRSPA, SREBF2, PPARA* and *PPARG*) and their numerous downstream lipolytic versus lipogenic genes. Many of these transcriptional regulators and miRNA-targeted lipolytic and lipogenic genes were identified in our recent contrasts of liver transcriptomes in embryos versus hatchling chicks [5].

## Conclusions

Our analyses of liver transcriptome during a fasting-refeeding perturbation immediately after hatching shows that *THRSPA* functions as the ultimate transcriptional regulator of lipogenesis and thermogenesis in the chicken. Our present integration of transcriptional profiling of liver in newly-hatched chicks during a fasting and re-feeding episode has revealed several differentially-expressed upstream regulators. These transcription factors are interactive and interdependent, which provides the *homeorhetic* mechanisms driven by reciprocal actions and interactions of nuclear factors that activate or inhibit response elements in promotors of other transcription factors. Interactions of few transcription factors and their more numerous target genes enable the switch in metabolism from a fasting (*lipolytic/ gluconeogenic*) state to the refed or fully-fed (*lipogenic/thermogenic*) state. This rapid *homeorhetic* shift of whole-body metabolism from a catabolic fasting state to the fed/refed anabolic state appears precisely orchestrated by a small number of ligand-activated transcription factors. These hepatic transcription factors favor either a fasting lipolytic state (*PPARA, NR3C1, FOX01, NFE2L2, SERTAD2, NR0B1* and *RXR*) or the fed lipogenic state (*THRSPA, SREBF2, PPARG, PPARD, CTNNB1* and *JUN*). These upstream regulators control of a variety of downstream genes encoding: metabolic enzymes, transporters, acute-phase response proteins, and clotting/immune factors. Several signaling factors were mapped to canonical metabolic and regulatory pathways (i.e., lipid metabolism, gluconeogenesis and glycolysis, growth factor signaling, and immune defense). Clearly, *THRSPA* has emerged as the predominant lipogenic and thermogenic transcription factor and most responsive gene found in liver of newly-hatched (D0-D4) chicks in the fed or refed state. Furthermore, the diminished growth that follows prolonged (48 h) fasting of newly-hatched chicks also illustrates the importance of early placement of day-old broiler chicks on feed (and water), before they deplete retained yolk lipids.

## Methods

### Experimental design

**Fig. 13:**
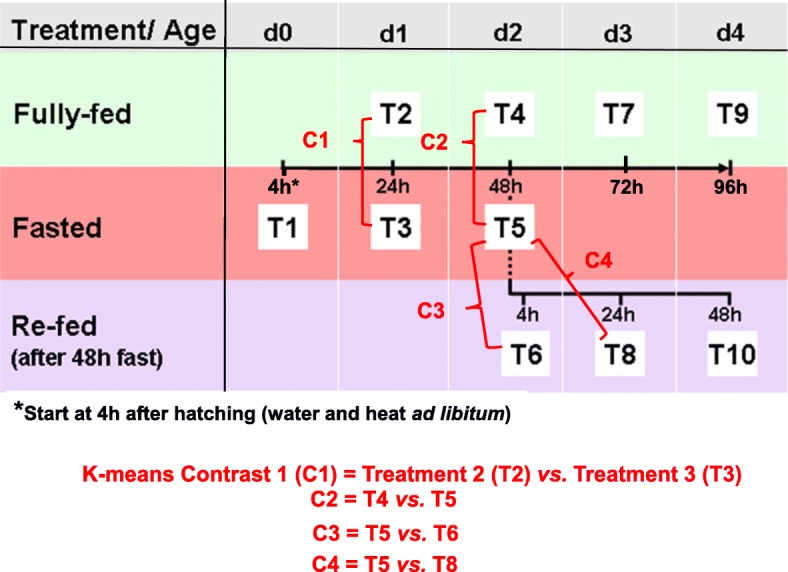
Experimental design of the fasting and re-feeding perturbation in newly-hatched broiler chicks. Five newly-hatched male chicks were randomly assigned to 10 treatment groups (T1-T10). The chicks assigned to control fully-fed groups (D1FED24h or T2, D2FED48h or T4, D3FED72h or T7, and D4FED96h or T9) were provided with a commercial starter ration and water ad libitum from hatching (D0) until the time of tissue sampling. Fasting groups (D0FAST4h or T1, D1FAST24h or T3, and D2FAST48h or T5) of chicks were brooded with no access to feed (start after hatchling) for 4, 24 and 48 h, respectively. Chicks in the REFED groups (D2REFED4h or T6, D3REFED24h or T8 and D4REFED48h or T10) were fasted for 48 h and subsequently re-fed for 4, 24 and 48 h, respectively, prior to the time of tissue sampling

A randomized block design was used to administer 10 treatment conditions (T1-T10), each containing five biological samples (5 cockerels), and taken during a bout of fasting and re-feeding in newly-hatched (D0-D4) broiler cockerels (Fig. 13.) Several experimental designs and statistical analyses (e.g., K-Means Clustering in Fig. 1) were used in the following sections and described accordingly.

#### Experimental chickens

Fertile eggs (Ross x Ross breed) were obtained from a commercial hatchery (Allen’s Hatchery, Seaford DE) and incubated in the Animal Facility, Department of Animal and Avian Sciences, University of Maryland (College Park, MD). The same experimental design was used earlier for our transcriptional and pathway analysis of the hypothalamus during a fasting and refeeding perturbation of newly-hatched (D0-D4) chicks [8]. Briefly, fertile eggs were incubated under standard conditions (37.5 °C and 60% relative humidity) with automatic rotation every hour and transferred to a hatching incubator on embryonic day 18 (E18), where E0 is defined as the start of incubation. All chicks (*N* = 50) used in this experiment were hatched within a 3–4-h period on E21 (D0) and given free access to water. After hatching (D0) chicks were randomly assigned to ten treatment groups, namely T1-T10 (Fig. 13). Only male chicks (cockerels) were used prevent confounding effects of sex. At hatching, control fed groups (T2, T4, T7 and T9; *n* = 20) were immediately provided ad libitum with water and a commercial starter ration until the time of tissue sampling on D1, D2, D3 and D4, respectively. After hatching, chicks in the T1, T3 and T5 groups (*N* = 15) were brooded with water freely available, but without access to feed (i.e., prolong-fasting) for 4, 24 and 48 h, respectively. Chicks in the T6, T8 and T10 groups (*N* = 15) were fasted for 48 h and subsequently re-fed for 4, 24 and 48 h, respectively, prior to tissue sampling. Male sex was visually verified upon visceral dissection and collection of liver samples. After collection of a 2 ml blood sample and euthanasia by cervical dislocation, liver samples were quickly excised, immediately snap frozen in liquid nitrogen, and then stored at − 80 °C until total RNA isolation for microarray and qRT-PCR analyses. Plasma samples were analyzed in duplicate for metabolite levels (glucose, non-esterified fatty acids (NEFA), and triglycerides). The care, maintenance and experimental use of hatchling chicks (*Gallus gallus*) were performed in accordance with the United States Department of Agriculture (USDA) guidelines on the Use of Agricultural Animals in Research and approved by the University of Maryland and the University of Delaware Agricultural Animal Care and Use Committees (AACUC).

#### Plasma glucose, non-esterified fatty acid (NEFA) and triglyceride assays

Circulating glucose, NEFA and triglyceride concentrations were determined in duplicate from plasma collected from five individual birds per treatment group (*N* = 50). Samples were stored at − 20 °C until the metabolite assays. Plasma glucose levels were determined using Autokit Glucose reagents (Wako Chemicals Richmond, VA) according to the manufacturer’s instructions. Plasma NEFA levels were determined using the Wako NEFA HR kit and triglyceride concentrations were determined using a L-Type TG H (Wako Chemicals Richmond, VA) following the assay kit’s instructions. All assays were optimized for microtiter plates and performed in a SpectraMax 190 microtiter plate reader (Molecular Devices, Sunnyvale, CA).

### Microarray analysis

#### Chicken long oligo microarrays

Fifty microarray slides printed with 20,676 (20.7 K) chicken oligonucleotides (oligo) were obtained from the Genomics Research Laboratory at the Steele Children’s Research Center at the University of Arizona (http://www.grl.steelecenter.arizona.edu/products.asp). Chicken long oligos (70-mers) for this genome-wide microarray were originally designed by ARK-Genomics (http://www.ark-genomics.org/microarrays/bySpecies/chicken/) from chicken ENSEMBL transcripts. The chicken oligo set [*Gallus gallus* (chicken) Roslin/ARK CoRe Array V1.0] was manufactured by Operon Technologies, Inc. The detailed description of the “Arizona” 20.7 K chicken oligo microarray is available at NCBI GEO (Platform GPL6049). The detailed annotation file of oligos printed on this microarray was obtained from European Animal Disease Genomics Network of Excellence (EADGENE) project [47]. Automated annotation of differentially-expressed oligo targets on the Arizona 20.7 K array was achieved using the “Oligo Arrays” function on our laboratory website (http://cogburn.dbi.udel.edu/index.html).

#### RNA isolation and dye labeling

Total RNA from frozen liver samples was isolated using an RNeasy Midi kit (Qiagen, Valencia, CA) following the manufacturer’s protocol. The quantity of total RNA was determined using a NanoDrop spectrophotometer (Wilmington, DE), and RNA quality was examined by microcapillary electrophoresis with a BioAnalyzer 2100 (Agilent, Wilmington, DE). RNA amplification was performed with an Amino Allyl MessageAmp™ II aRNA Amplification Kit according to manufacturer’s instruction (Ambion, Austin, TX). A single round of RNA amplification was conducted with 4 μg of total RNA. In vitro transcription (IVT) of amino allyl-modified aRNA was performed for 14 h at 37 °C. The integrity of aRNA was determined by a BioAnalyzer using the RNA 6000 Nano Assay kit and the concentration of aRNA with a NanoDrop ND-1000 spectrophotometer prior to the aRNA-dye coupling reaction. The dye labeling was performed according to the instructions for dye coupling and labeled aRNA cleanup with the Amino Allyl MessageAmp™ II aRNA Amplification kit. Briefly, twenty micrograms of aRNA was dried down in a SpeedVac concentrator, reconstituted in 9 μl of coupling buffer and mixed with 11 μl of prepared fluorescent dyes (60 μg aliquot of either Alexa 555 or Alexa 647 dissolved in DMSO). The coupling reaction took place in the dark for 1 hour at room temperature. Purified labeled aRNA was determined for concentration and dye incorporation using a NanoDrop and dye incorporation efficiency was calculated with a web tool (www.ambion.com/tools/dye). The balanced-block hybridization design used for the 50 oligo arrays used in the present study is provided in Additional file 9.

#### Hybridization and scanning of microarrays

The chicken oligo microarrays were baked at 90 °C for 90 min prior to use. The slides were pre-treated for 45 min at 42 °C in pre-hybridization solution (5x SSC, 0.1% SDS and 1% BSA) and followed by dipping in 2x SSC for 5 min and then in 0.2x SSC for 5 min. The slides were dried by centrifugation (5 min at 1000 x g). An aliquot of five micrograms of purified labeled aRNA samples was fragmented in 1x fragmentation buffer (Ambion, Austin, TX) for 15 min at 70 °C. The reaction was then terminated by adding stop solution and dried down in a SpeedVac. Each fragmented aRNA sample was reconstituted in 30 μl of pre-warmed DIG Easy Hyb solution (Roche Diagnostics Corporation, Indianapolis, IN). All reference aRNA samples within the same hybridization day were pooled together, equally divided in 30 μl aliquots and co-hybridized with each target sample on a slide. The target and reference aRNA samples were mixed with 2.5 μl of 10 mg/ml yeast tRNA and 2.5 μl of 10 mg/ml salmon testes DNA (Sigma, Louis, MO). The reference/target mixture (65 μl) was denatured for 2 min at 94 °C, cooled at room temperature, carefully loaded onto the middle of a pre-treated slide held in a hybridization chamber (Corning #2551, Lowell, MA) and overlaid with a 22 × 65 mm glass coverslip (LifterSlip; Erie Scientific, Portsmouth, NH). The sealed hybridization chambers were incubated overnight (14–16 h) at 42 °C in a water bath covered with a light-tight box. On the following day, the cover slips were flushed off in wash solution #1 (1x SSC, 0.2% SDS, and 0.5% DTT) and slides were sequentially washed with pre-warmed wash solution #1 at 42 °C for 10 min, then washed in solution #2 (0.1 x SSC, 0.2% SDS, and 0.5% DTT) for 5 min at room temperature, and finally washed in solution #3 (0.1 x SSC and 0.5% DTT) for 1 min at room temperature. The slides were subsequently rinsed in MilliQ-purified water and dried by centrifugation. The slides were stored in individual 50 ml tubes covered in aluminum foil and flooded with pure nitrogen gas until scanning. For each slide, a high-resolution TIFF image file was generated using a GenePix 4000B microarray scanner and analyzed with GenePix Pro V4.1 software (Axon Laboratories, Palo Alto, CA). The TIFF images were acquired at 10 μm resolution by simultaneous scanning at two wavelengths [532 nm (Alexa 555) and 635 nm (Alexa 647)]. The PMT count ratio (635/532) was adjusted to 1 during preview at low resolution scanning. All slides were manually checked for quality and all spots with inadequacies in signal, background or morphology were eliminated. The image analysis results were merged with Excel files (in GPR format) containing clone identification, spot location on slide, and most current gene annotation based on BLASTN or BLASTX score. The GPR files from the 50 microarray scans were then used for normalization and statistical analysis to determine differential gene expression.

#### Normalization and statistical analysis of microarray data

The processing, normalization and determination of differential gene expression was completed using the Linear Models for Microarrays (LIMMA) program [48], as previously described for a reference RNA hybridization design [49]. Briefly, log2 transformed median intensity values for each dye (Alexa Flour™ 555 or Alexa Flour™ 647) were normalized using a LOWESS transformation [50], without background correction [51, 52]. The Benjamini-Hochberg procedure [53] was used to control experiment-wise false discovery rate (FDR ≤ 0.05) associated with multiple testing (FDR adjusted *P* ≤ 0.05). Gene expression represents the log2 of normalized dye ratios (sample aRNA-green/reference aRNA-red). The log2 FC was further scaled to have the same median-absolute deviation [54]. As described earlier [49], the LIMMA output was statistically analyzed using a linear mixed model in SAS (Statistical Analysis System, Cary NC). Mixed models in SAS [55, 56] were used to account for biological and technical variation (dye, day of hybridization) across the 50 oligo microarrays and to determine differential expression of a gene possessing a FDR-adjusted *p*-value (*P* ≤ 0.05). Forty-five pairwise contrasts were made across the 10 treatment conditions, where gene expression values represent the log2 fold-change (FC) [57] of treatment contrasts (e.g., T4/T5 or D2FED48h/D2FAST48h). Four pairwise contrasts (C1 = T2/T3; C2 = T4/T5; C3 = T5/T6; C4 = T5/T8) were used for K-means cluster analysis to identify clusters of functionally-related genes (see Fig. 13; Additional file 1). Lists of differentially-expressed genes (DEGs) from pairwise contrasts were initially annotated using the “Oligo Arrays” function on our laboratory website [58], which was specifically designed for the horizontally-printed Arizona 20.7 K Chicken Oligo Array (NCBI GEO Platform GPL6049). Finally, lists of DEGs from each contrast were used as input for Ingenuity Pathway Analysis and further functional annotation. This minimum information about a microarray experiment (MIAME)-compliant dataset was deposited in the NCBI GEO database under accession number GSE9745.

#### Hierarchical cluster analysis

A preliminary hierarchical cluster analysis of DEGs was conducted using GeneSpring GX software (Agilent Technologies, Santa Clara, CA) to initially visualize differences in gene expression patterns across 50 chick liver transcriptomes during an episode of fasting and refeeding (D0-D4). This dataset was used to generate a heat map of differentially expressed gene (DEG) across the first 4 days of life and under the metabolic perturbation of fasting and refeeding (10 treatment conditions). This dataset from unsupervised hierarchical clustering was only used for preliminary visual analysis of gene expression patterns during fasting and refeeding (see Fig. 2a).

#### K-means cluster analysis

K-means clustering was used for visualization of gene expression patterns in response to fasted vs. refeed conditions (see Fig. 2b; Additional file 1). Treatment contrasts (C1-C4) represent log2 ratios of four pair-wise comparisons [C1 = D1FED vs. D1FAST24h, C2 = D2FED vs. D2FAST48h, C3 = D2FAST48h vs. D3REFED4h, and C4 = D2FAST48 vs. D3REFED24h]. Contrasts C1 and C2 have positive or negative log2 fold-change (FC) values, which represent down-regulation or up-regulation by fasting, respectively. Likewise, FC for contrasts C3 and C4 indicate either down-regulation or up-regulation due to re-feeding for 4 h or 24 h (after a 48 h fast), respectively. These log2 FC values were multiplied by − 1 to make the relative expression (log2 FC) either positive for up-regulation or negative for down-regulation of gene expression.

### Functional analysis of DEGs using ingenuity pathway analysis (IPA)

Ingenuity® Pathway Analysis (IPA) was used for functional annotation and mapping of DEGs to canonical pathways, biological processes and gene interaction networks. IPA Fisher’s Exact Test is used by IPA to test for significance (*P* ≤ 0.05) and over-representation of DEGs in canonical pathways and biological processes. The DEGs accepted by IPA are considered as “Analysis Ready” (AR)-DEGs, if the gene is curated and annotated in the Ingenuity® Knowledge Base, which is mainly curated from the mammalian biomedical literature and devoid of many avian-specific genes. A combination of the chicken gene symbol (primary gene ID) and the NCBI RefSeq Protein ID (secondary gene ID) was used to maximize the number of chicken genes DEGs accepted by IPA as AR-DEGs. However, almost a quarter of DEGs with a chicken-specific RefSeq Protein ID were rejected by IPA software (see Additional file 2). The Ingenuity® Upstream Regulator Analysis within IPA was used to identify major transcription factors and to predict either activation or inhibition of direct target genes by their upstream regulators.

### Quantitative reverse transcription-PCR (qRT-PCR) analysis and verification of DEGs

An aliquot of each total RNA sample from the 50 liver samples (10 treatments with 5 biological replicates per treatment) was used for qRT-PCR analysis to verify differential gene expression. Sixteen differentially-expressed genes (DEGs) identified by microarray analysis, were used for qRT-PCR analysis, plus an additional four “invariant” genes that were used for normalization of qRT-PCR expression levels (Additional file 10). Selection of the 16 “candidate” genes was based on prior knowledge of their involvement in lipid metabolism. In additional, a panel of four invariant genes (*ATPCL*, *COX7A2L, GAPDH* and *PRL14*) was included in the qRT-PCR analysis for normalization of qRT-PCR expression. First-strand cDNA was synthesized using 1 μg of total RNA, oligo-dT, random hexamer primers and SuperScript III reverse transcriptase kit (Invitrogen Life Technologies, Carlsbad CA). Primers used for qPCR were designed using Primer Express (Applied Biosystems, Foster City, CA). The qRT-PCR assay was performed for each sample in duplicate wells using Power SYBR green PCR master mix (Applied Biosystems, Foster City, CA) and a gene-specific primer-pair (Sigma-Genosys, Woodlands, TX) on an ABI Prism Sequence Detection System 7900HT. Biogazelle qbase+ software [59] was used for pre-processing of raw cycle threshold (Ct) data and the geNorm procedure was used for normalization of relative gene expression levels. The expression stability of candidate genes and a panel of invariant “housekeeping” genes was based on the geNorm (M) and coefficient of variation (CV) values across duplicate measurements of the 50 liver RNA samples. Ribosomal protein L14 (*RPL14*) and cytochrome c oxidase subunit VIIa polypeptide 2-like (*COX7A2L*) were selected as optimal co-reference genes. The PROC GLM in Statistical Analysis System (SAS, v.9.4; Cary NC) was used to analyze differences among the 10 treatment groups and control for multiple comparisons using the option ADJUST = TUKEY. The Pearson’s correlation function in Excel was used to compare normalized expression levels of “candidate” DEGs identified by microarray analysis with normalized qRT-PCR expression levels.

## Supplementary information

**Additional file 1: **K-means clusters identified from four pairwise contrasts of fasting and refeeding conditions. A Microsoft Excel file containing two worksheets named: “K-means clusters_FDR adj *P*≤0.05” and “Plots of K-Means Clusters (C0-C9)”. The first worksheet provides an annotated list of the 196 DEGs assigned to 10 clusters (C0-C9) of functionally-related hepatic genes. Information is provided on each DEG including the RIGG oligo ID, gene symbol, gene description, log2 fold-change (FC) of pairwise contrasts of fasting (C1 and C2) and refeeding (C3 and C4) conditions, and the cluster number. The second worksheet contains a PowerPoint figure composed of 10 K-Means Clusters that differentially respond to fasting and refeeding conditions.

**Additional file 2: **Comparison of DEGs identified by microarray analysis versus *“*Analysis Ready” (AR)-DEGs accepted by IPA. A Microsoft Excel file containing a single worksheet “ IPA rejection of DEGs”. This worksheet list the number of chicken DEGs (FDR adj. *P* ≤ 0.05) identified by microarray analysis and the number of “Analysis Ready” (AR)-DEGs recognized by the Ingenuity Knowledge Base and used for Ingenuity Pathway Analysis (IPA). On average, 22.5% of the chicken microarray DEGs identified with a valid RefSeq ID were rejected by IPA.

**Additional file 3: **Lists of DEGs from five meaningful pairwise contrasts of fasting and refeeding conditions. A Microsoft Excel file containing five worksheets of AR-DEGs (FDR adjusted *P* ≤ 0.05) identified from pairwise contrasts. Each worksheet provides a list of AR-DEGs identified by pairwise treatment contrasts including the gene symbol, Entrez gene name, log2 expression ratio, and gene/protein ID number.

**Additional file 4: **Over-represented canonical and functional pathways identified by IPA in the D2FED48h vs D2FAST48h contrast. A Microsoft Excel file containing eight worksheets of AR-DEGs (FDR adjusted *P* ≤ 0.05) mapped to over-represented canonical and functional pathways. Each worksheet provides the gene symbol, Entrez gene name, log2 ratio, cellular location and type of gene.

**Additional file 5: **Over-represented canonical and functional pathways identified by IPA in the D2FAST48h vs D3REFED24h contrast. A Microsoft Excel file containing eight worksheets of AR-DEGs (FDR adjusted *P* ≤ 0.05) mapped by IPA to over-represented canonical and functional pathways. Each worksheet provides the gene symbol, Entrez gene name, log2 ratio, cellular location and type of gene.

**Additional file 6: **Over-represented canonical and functional pathways identified by IPA in the D2FAST48h vs D4REFED48h contrast. A Microsoft Excel file containing eight worksheets of AR-DEGs (FDR adjusted *P* ≤ 0.05) mapped to over-represented canonical and functional pathways. Each worksheet provides the gene symbol, Entrez gene name, log2 ratio, cellular location and type of gene.

**Additional file 7: **Four additional genes used for qRT-PCR analysis. A PowerPoint figure showing qRT-PCR analysis of two candidate genes [FAT atypical cadherin 1 (*FAT1*); glyceraldehyde-3-phosphate dehydrogenase (*GAPDH*)] and two invariant genes [cytochrome c oxidase subunit 7A2 like (*COX7A2L*); ribosomal protein L14 (*RPL14*)]. Both *COX7A2L* and *RPL14* were used by geNorm software for normalization of gene expression as determined by qRT-PCR analysis. Nutritional state is indicated by bar color, where green = fed, red = fasted, and blue = refed conditions. Values represent least-square means (LSM) and their standard error (LSE) of normalized expression levels of five cockerels (biological replicates) analyzed in duplicate. Expression levels, determined by qRT-PCR analysis, were normalized using the geNorm procedure in qBase software [59]. Values possessing different superscripts are significantly different as determined by analysis of variance (ANOVA) using the general linear models (GLM) procedure in Statistical Analysis System (SAS) software and with mean separation using Tukey’s Studentized Range Test.

**Additional file 8: **Pearson’s Correlation Analysis of gene expression levels of 10 DEGs determined by genome microarray and verified by qRT-PCR analyses. A Microsoft Excel file containing the average normalized expression level (log2 ratio) of 10 DEGs as determined by both microarray analysis and qRT-PCR analysis. The Pearson’s correlation coefficient (r = 0.970; *P* ≤ 0.01, 8 degrees of freedom) indicates a highly significant correlation between gene expression levels obtained from both microarray and qRT-PCR analyses.

**Additional file 9:** Microarray reference RNA hybridization design. A Microsoft Excel file containing a single worksheet “20.7 K Array Hybridization Design”. A total of 50 Arizona 20.7 K Chicken Oligo Arrays (NCBI GEO Platform # GPL6049) were used in a reference RNA hybridization design. Each biological sample (liver RNA sample) was labelled with Alexa Flour® 555 (green) dye. An aliquot of the pooled reference RNA sample was labelled with Alexa Flour® 647 (red) dye and these were hybridized together on one of the 20.7 K oligo microarrays. The 50 microarrays were assigned to 10 fasting-refeeding treatment groups with five biological replicates (liver RNA from five cockerels). A balanced block design was used for hybridization of 10 microarrays across 5 consecutive days, where one liver RNA sample represented each of the 10 treatment groups on each hybridization day (10 arrays/day).

**Additional file 10: **Information on primers used for qRT-PCR verification of differential gene expression. A Microsoft Excel file containing a single worksheet “Primers qRT-PCR Assay”. This table provides information on the design of qRT-PCR primers for 20 chicken genes. The Roslin Institute *Gallus gallus* (RIGG) oligo ID number, NCBI Entrez Gene ID, gene symbol, gene description, and the 5′-3′ sequence for forward and reverse primers used for qRT-PCR analysis are provided. Although four “invariant genes” were included, only two genes were acceptable by qBase software for use in normalization of qRT-PCR expression levels.

## Data Availability

The files containing minimum information about a microarray experiment (MIAME)-compliant microarray experiment are available from the NCBI GEO database under accession number GSE9745. Additional data generated or analyzed during this study are included within this published article and supplementary files. Multiple worksheets in Additional file 3 provide lists of DEGs found in four meaningful pair-wise contrasts of fed, fasted and refed chicks from statistical analysis of the microarray data.

## Abbreviations

*ACSL5*: Acyl-CoA synthetase long chain family member 5
*ADIPOR2*: Adiponectin receptor 2
*AGO1*: Argonaute 1
*ANGPTL3*: Angiopoietin like 3
ANOVA: Analysis-of-variance
*ATF4*: Activating transcription factor 4a
*CREB2*: cAMP-response element binding protein 2
Ct: Average cycle time
*CTNNB1*: Catenin beta 1
D0-D4: Age, Day 0-Day 4
DEGs: Differentially-expressed genes
*DHCR7*: 7-dehydrocholesterol reductase
*DIO2*: Type 2 deiodinase
*F11*: Coagulation factor XI
*F2*: Coagulation factor II, thrombin
*F9*: Coagulation factor IX
*FADS2*: Fatty acid desaturase 2
FC: Fold change
FDR: False discovery rate
*FKBP5*: FK506 binding protein 5
GCR: Glucocorticoid receptor
GEO: Gene Expression Omnibus
GLM: General linear model
*IGFBP2*: Insulin-like growth factor binding protein 2
IPA: Ingenuity Pathway Analysis
*JUN*: Jun proto-oncogene, AP-1 transcription factor subunit
LIMMA: Linear Models for Microarrays
*lncRNA*: Long non-coding RNA
LSD: Least significant difference
*ME1*: Malic enzyme 1
MIAME: Minimum information about a microarray experiment
*miRNA*: microRNA
*MST1*: Macrophage stimulating 1
*MUSTN1*: Embryonic nuclear protein 1
NCBI: National Center for Biotechnology Information
*PANK1*: Pantothenate kinase 1
*PROC*: Protein C, inactivator of coagulation factors Va and VIIIa
*PROZ*: Protein Z, vitamin K dependent plasma glycoprotein
qRT-PCR: Quantitative reverse transcriptase polymerase chain reaction
*RARRES2*: Retinoic acid receptor responder 2
RIN: RNA integrity number
RNA: Amplified RNA
*RXR*: Retinoid X receptor
SAS: Statistical Analysis System
*SERPINC1*: Serpin family C member 1
*SERTAD2*: SERTA domain containing 2
*SIRT1*: Sirtuin 1
*SIRT5*: Sirtuin 5
*THRSPA*: Thyroid hormone-responsive Spot 14 protein, alpha
*UPP2*: Uridine phosphorylase 2
