# Supplementary material for: Transcriptome analyses of liver in newly-hatched chicks during the metabolic perturbation of fasting and re-feeding reveals THRSPA as the key lipogenic transcription factor
Source: BMC Genomics. 2020 Jan 31;21:109. doi: 10.1186/s12864-020-6525-0 (PMC6995218; doi:10.1186/s12864-020-6525-0)
Supplement: Supplementary file 7 — Additional file 7: Four additional genes used for qRT-PCR analysis. A PowerPoint figure showing qRT-PCR analysis of two candidate genes [FAT atypical cadherin 1 (FAT1); glyceraldehyde-3-phosphate dehydrogenase (GAPDH)] and two invariant genes [cytochrome c oxidase subunit 7A2 like (COX7A2L); ribosomal protein L14 (RPL14)]. Both COX7A2L and RPL14 were used by geNorm software for normalization of gene expression as determined by qRT-PCR analysis. Nutritional state is indicated by bar color, where green = fed, red = fasted, and blue = refed conditions. Values represent least-square means (LSM) and their standard error (LSE) of normalized expression levels of five cockerels (biological replicates) analyzed in duplicate. Expression levels, determined by qRT-PCR analysis, were normalized using the geNorm procedure in qBase software [59]. Values possessing different superscripts are significantly different as determined by analysis of variance (ANOVA) using the general linear models (GLM) procedure in Statistical Analysis System (SAS) software and with mean separation using Tukey’s Studentized Range Test. [file 12864_2020_6525_MOESM7_ESM.pptx]

## Slide 1
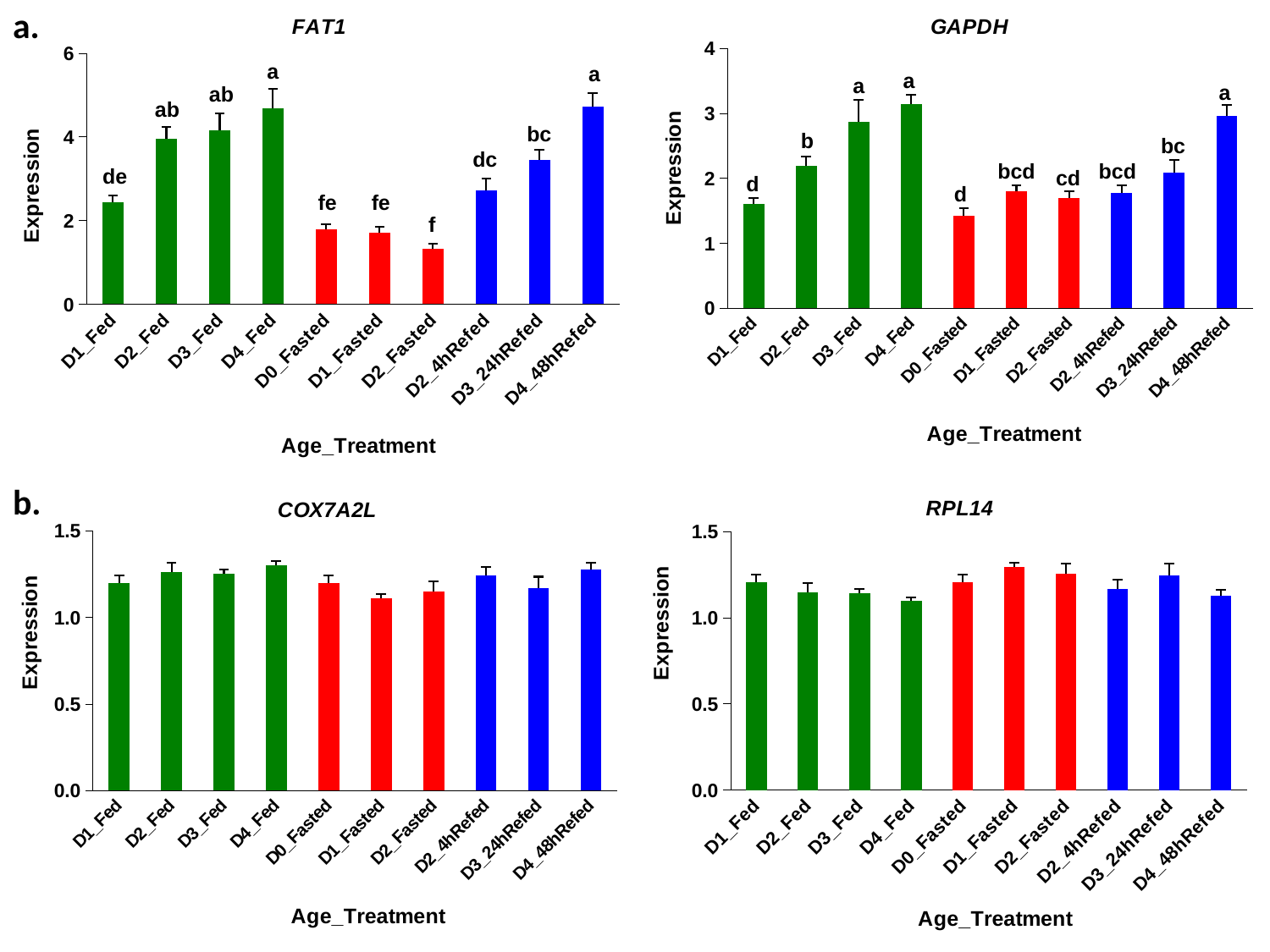

a.
### Chart: GAPDH
| Category | |
|---|---|
| D1_Fed | 1.61166667 |
| D2_Fed | 2.19333333 |
| D3_Fed | 2.86333333 |
| D4_Fed | 3.14166667 |
| D0_Fasted | 1.42 |
| D1_Fasted | 1.79666667 |
| D2_Fasted | 1.69333333 |
| D2_4hRefed | 1.77 |
| D3_24hRefed | 2.09333333 |
| D4_48hRefed | 2.95333333 |a
a
a
b
bc
bcd
bcd
cd
d
d
### Chart: FAT1
| Category | |
|---|---|
| D1_Fed | 2.44 |
| D2_Fed | 3.94833333 |
| D3_Fed | 4.15333333 |
| D4_Fed | 4.69 |
| D0_Fasted | 1.79166667 |
| D1_Fasted | 1.71333333 |
| D2_Fasted | 1.33333333 |
| D2_4hRefed | 2.71666667 |
| D3_24hRefed | 3.44333333 |
| D4_48hRefed | 4.71666667 |a
a
ab
ab
bc
dc
de
fe
fe
f
b.
### Chart: RPL14
| Category | |
|---|---|
| D1_Fed | 1.20333333 |
| D2_Fed | 1.14833333 |
| D3_Fed | 1.14333333 |
| D4_Fed | 1.09833333 |
| D0_Fasted | 1.205 |
| D1_Fasted | 1.29166667 |
| D2_Fasted | 1.255 |
| D2_4hRefed | 1.16666667 |
| D3_24hRefed | 1.245 |
| D4_48hRefed | 1.125 |
### Chart: COX7A2L
| Category | |
|---|---|
| D1_Fed | 1.19666667 |
| D2_Fed | 1.26 |
| D3_Fed | 1.25333333 |
| D4_Fed | 1.30333333 |
| D0_Fasted | 1.19666667 |
| D1_Fasted | 1.11166667 |
| D2_Fasted | 1.15166667 |
| D2_4hRefed | 1.24 |
| D3_24hRefed | 1.16833333 |
| D4_48hRefed | 1.27833333 |
